# A genome-wide association study of seed composition traits in wild soybean (*Glycine soja*)

**DOI:** 10.1186/s12864-016-3397-4

**Published:** 2017-01-05

**Authors:** Larry J. Leamy, Hengyou Zhang, Changbao Li, Charles Y. Chen, Bao-Hua Song

**Affiliations:** 1 Department of Biological Sciences, the University of North Carolina at Charlotte, Charlotte, NC 28223 USA; 2 Double Haploid Optimization Group, Monsanto Company, Chesterfield, MO 63017 USA; 3 Department of Crop, Soil and Environmental Sciences, Auburn University, Auburn, AL 36849 USA

**Keywords:** Quantitative trait loci, Fatty acids, Protein, Oil, Candidate genes, GWAS

## Abstract

**Background:**

Cultivated soybean (*Glycine max*) is a major agricultural crop that provides a crucial source of edible protein and oil. Decreased amounts of saturated palmitic acid and increased amounts of unsaturated oleic acid in soybean oil are considered optimal for human cardiovascular health and therefore there has considerable interest by breeders in discovering genes affecting the relative concentrations of these fatty acids. Using a genome-wide association (GWA) approach with nearly 30,000 single nucleotide polymorphisms (SNPs), we investigated the genetic basis of protein, oil and all five fatty acid levels in seeds from a sample of 570 wild soybeans (*Glycine soja*), the progenitor of domesticated soybean, to identify quantitative trait loci (QTLs) affecting these seed composition traits.

**Results:**

We discovered 29 SNPs located on ten different chromosomes that are significantly associated with the seven seed composition traits in our wild soybean sample. Eight SNPs co-localized with QTLs previously uncovered in linkage or association mapping studies conducted with cultivated soybean samples, while the remaining SNPs appeared to be in novel locations. Twenty-four of the SNPs significantly associated with fatty acid variation, with the majority located on chromosomes 14 (6 SNPs) and seven (8 SNPs). Two SNPs were common for two or more fatty acids, suggesting loci with pleiotropic effects. We also identified some candidate genes that are involved in fatty acid metabolism and regulation. For each of the seven traits, most of the SNPs produced differences between the average phenotypic values of the two homozygotes of about one-half standard deviation and contributed over 3% of their total variability.

**Conclusions:**

This is the first GWA study conducted on seed composition traits solely in wild soybean populations, and a number of QTLs were found that have not been previously discovered. Some of these may be useful to breeders who select for increased protein/oil content or altered fatty acid ratios in the seeds. The results also provide additional insight into the genetic architecture of these traits in a large sample of wild soybean, and suggest some new candidate genes whose molecular effects on these traits need to be further studied.

**Electronic supplementary material:**

The online version of this article (doi:10.1186/s12864-016-3397-4) contains supplementary material, which is available to authorized users.

## Background

Cultivated soybean (*Glycine max* (L.) Merr.) is a major agricultural crop that provides a crucial source of edible protein and oil [1]. Although the seed protein composition typically is about double that of oil, soybean produces over half of the world’s vegetable oil (http://www.soystats.com). Soybean oil is comprised almost entirely of two saturated (palmitic and stearic acid), and three unsaturated fatty acids (oleic, linoleic, and linolenic acid). The relative proportions of these fatty acids determine the overall quality of the oil, with decreased amounts of saturated palmitic acid and increased amounts of unsaturated oleic acid considered optimal for human cardiovascular health [2, 3]. Minimal amounts of linoleic and linolenic acid also are beneficial because partial hydrogenation of soybean oil routinely done to improve the oxidative stability of these fatty acids leads to the production of undesirable *trans*-fats [4].

Given the enormous agricultural importance of seed composition traits in soybean, it is not surprising that there have been a number of studies aimed at understanding their genetic basis [5–7]. Much of our genetic knowledge has come from discovery of quantitative trait loci (QTLs) located at various sites on all of the 20 chromosomes throughout the soybean genome that affect one or more of these traits [5, 6, 8–10]. Many of these QTLs were discovered through linkage mapping that requires F_2_, backcross, or recombinant inbred populations derived from original biparental crosses. With this approach, therefore, any QTLs found are limited to those whose alleles differ in the progenitor populations. A perhaps even greater difficulty with bi-parental linkage mapping is that the confidence intervals for the QTLs uncovered typically are quite wide (often 20 cM or more) because of extensive regions of linkage disequilibrium (LD) in the populations analyzed [11]. These large genomic regions may contain many underlying genes, making the search for putative candidates difficult.

Especially with the recent availability of large numbers of genomic markers in various taxa, genome-wide association studies (GWAS) increasingly have been used to search for QTLs affecting various traits [12, 13]. This approach can be applied to outbred or wild populations that have experienced extensive recombination resulting in shorter LD segments and therefore increased resolution of marker-phenotype associations. GWA studies sometimes can produce false negative results because of potential confounding factors such as population structure and cryptic relatedness [14, 15], however, but current statistical procedures adjust for these factors [16]. A number of GWA studies have been successfully applied to soybean seed composition traits [7, 17–21].

While this work on soybean has proceeded, to our knowledge no comparable GWA study has been conducted on seed composition traits solely in wild soybean (*Glycine soja* Sieb & Zucc.), the progenitor of cultivated soybeans. The level of genetic variability in soybean cultivars has been considerably reduced from that in *G. soja* [22–26] and therefore GWA analyses of this wild species may be expected to yield some novel QTLs for seed composition traits. We conducted such an analysis of protein, oil and fatty acid content in 570 wild soybean accessions genotyped with nearly 30,000 SNPs. We document a number of SNPs significantly associated with these traits, with some suggestions for candidate genes important for the seed composition trait variation.

## Methods

### Plant material and traits measured

The source material for the analysis originated from over 600 *G. soja* accessions in the USDA Soybean Germplasm Collection representing maturity groups V through IX. These accessions were originally collected from various sites primarily in South Korea and Japan although also from a few sites in China (Additional file 1). All seeds from these accessions were soaked for 15 min in concentrated sulfuric acid, rinsed in water and then air-dried prior to planting. One replicate from each accession was planted on May 19^th^, 1998 in hill plots in an open field in Stoneville, MS (Lat. 33°26’N), using a completely randomized design (CRD). One replicate from each accession also were planted on May 14, 1999 in the same plots using a randomized complete block (RCB) design. The wild soybean plants were allowed to mature at which time seeds were harvested and used to quantify protein, oil and fatty acid content. In the analysis we used the average of the two replicate values for each accession. The total number of individuals available was 570.

Seed composition measurements included protein and oil concentration and the concentration of five fatty acids: palmitic, stearic, oleic, linoleic and linolenic. Nitrogen content of whole seeds was determined with a LECO FP-428 Nitrogen Determinator (LECO Corp, St. Joseph MI). A 6.25 conversion factor was used to calculate protein concentration on a dry weight basis. Oil concentration (dry weight basis) of whole seeds was determined with a 5 MHz nuclear magnetic resonance spectrometer (Newport Oxford Instruments, Newport Pagnell, England). Fatty acid methyl esters were prepared from chloroform/hexane/methanol (8:5:2, v/v/v) extracts of crushed seed by transmethylation with sodium methoxide. Fatty acid composition was determined with a Hewlett-Packard 5890-II (Palo Alto, CA) gas chromatograph equipped with dual flame ionization detectors, and a 0.53 mm x 30 m AT-Silar capillary column (Alltech Associates, Deerfield, IL). Authentic fatty acids were used for calibration. Protein and oil were expressed as a percentage of the total seed content whereas all fatty acids were expressed as a percentage of the oil content.

### Genotyping and quality control

SNP data for the 570 accessions were retrieved from the publically-available soybase website (http://soybase.org/snps/), which were genotyped using the Illumina SoySNP50k iSelect BeadChip (Illumina, San Diego, CA. USA) containing a total of 52,041 SNPs as previously described [25, 27]. The SNP alleles were called using the GenomeStudio Genotyping Module v1.8.4 (Illumina, Inc. San Diego, CA). SNPs without a known physical position on any of the 20 chromosomes were excluded from further analyses. Further, for the genome-wide association analysis described below, we filtered the data by removing SNPs with missing rates >10%, minor allele frequencies < 0.05, and those existing in minor states so that only two alleles were segregating at each SNP locus. Heterozygote SNPs were also treated as missing since they were rare (<2%) and wild soybeans reproduce primarily by selfing. These adjustments reduced the number of SNPs used in the analysis to 29,969.

### Linkage disequilibrium estimation

We used the filtered SNP data to calculate linkage disequilibrium (LD) across the wild soybean genome with the TASSEL program, version 5 [28]. TASSEL produced two measures of LD: squared correlations (*r* ^2^s) and standardized disequilibrium coefficients (*D*’s). For various Kb distance intervals, we derived means of the distances between SNP pairs and then plotted these means against *r* ^2^ values to visualize the rate of LD decay.

### Preliminary statistical analysis

We first inspected the distributions of the phenotypic values for the seven traits and found that they were skewed for oleic and linoleic acid but approximately normal for the other five traits. We did not transform the values for any of the traits because our sample size was reasonably large [29]. We calculated basic statistics, including means and standard deviations, for these traits in the total sample of 570. In addition, Pearson correlations were calculated for each pair of traits, and their significance assessed by the false discovery rate procedure [30]. We also conducted one-way ANOVAs for each of the seven traits to test whether those originally from South Korea differed from those from Japan. We restricted this geographic analysis to these two countries because they comprised 534 of the 549 accessions whose locations were known.

### Genome-wide association analysis

We used TASSEL to test for the association of the seven traits with each SNP across the wild soybean genome. For each trait we first ran a general linear model (GLM) and then a compressed mixed linear model (CMLM) that included a kinship matrix (**K)** to account for familial relatedness. We also ran CMLMs that included the **K** matrix as well as the first 3 (**Q3**), 10 (**Q10**), 25 (**Q25)** or 50 (**Q50**) principal components (PCs) derived from a principal components analysis of the SNP data. The PC values were treated as fixed covariates in these models and were used to adjust for population structure. From the results for each of these models, we generated quantile-quantile (QQ) plots of the observed versus expected *p*-values at each SNP. For each trait, the model chosen for eventual analysis was that determined by the distribution of the QQ plot as well as its associated genomic inflation value (λ). Generally those plots that yielded λ values closest to 1.00 were considered optimal.

All probabilities generated in the association runs were transformed by –log_10_ *P*, and the highest scores on each chromosome were inspected to determine whether they reached a significance threshold. We calculated this threshold by first estimating the total number of independent SNPs following the method of Li and Ji [31]. This number was 11,149, considerably below the total number of SNPs used (29,969) because many of them were correlated due to linkage disequilibrium. The 5% genome-wide threshold therefore was established at a probability of 0.05/11,149 = 4.48 x 10^−6^, equivalent to a –log_10_ *P* score of 5.348. In addition, we considered a probability of 0.63/11,149 = 5.65 x 10^−5^ (-log_10_ *P* = 4.248) as being suggestive of a SNP/trait association. These 0.05 and 0.63 values are widely accepted thresholds for significant and suggestive QTLs [32]. We used conventional Manhattan plots to help with visualization of SNPs reaching either of these thresholds.

For all SNPs reaching at least the suggestive level of association, we tallied estimates of their allelic effects from TASSEL. These effects estimate the difference between the average phenotypic values of the two homozygotes. TASSEL also computed SNP *r* ^2^ values that we multiplied by 100 to estimate the percentage contribution of each SNP to the total phenotypic variation in each trait. We also calculated heritabilities for each trait across the entire genome from the ratio of the genetic variance to the total of the genetic and residual variance REML estimates obtained through the Efficient Mixed-Model Association (EMMA) algorithm in TASSEL.

### QTL and candidate gene search

Once all significant SNPs were identified, we used the soybean reference genome, SoyBase (www.soybase.org) to search for all genes located in the regions extending 50 Kb on each side of the SNPs. Any of these genes were regarded as potential candidate genes. We also used SoyBase to search for any QTLs in similar locations that previously have been found to affect the protein/oil or fatty acid traits.

## Results

### Wild soybean traits

Protein comprised nearly one-half (48%) of the total dry weight of the wild soybean seeds in our sample whereas the percentage of oil was 11% (Table 1). The three unsaturated fatty acids (oleic, linoleic, and linolenic acid) made up fully 83% of the content of the oil, with the remaining 17% contributed by the saturated fatty acids (palmitic and stearic acid). The coefficients of variation show that protein and linoleic acid are the least variable traits whereas oleic acid is the most variable. Correlations among the seven traits are mostly low to moderate in magnitude with the exception of the high, negative associations of oleic acid with both linoleic and linolenic acid. Nearly all (19/21) of the correlations are statistically significant (*P* < 0.05).

**Table 1:** Basic statistics for the seven traits in the wild soybean population (*n* = 570)

	Mean	StDev	CV	Oil	Palmitic Acid	Stearic Acid	Oleic Acid	Linoleic Acid	Linolenic Acid
Protein	48.10	2.626	5.46	−0.36*	0.23*	0.14*	0.22*	−0.26*	−0.17*
Oil	11.02	1.185	10.75		−0.20*	−0.04	0.30*	0.01	−0.34*
Palmitic Acid	12.90	1.020	7.91			0.33*	0.25*	−0.41*	−0.38*
Stearic Acid	3.89	0.466	11.98				0.36*	−0.30*	−0.49*
Oleic Acid	14.88	3.673	24.68					−0.78*	−0.76*
Linoleic Acid	54.27	2.686	4.95						0.28*
Linolenic Acid	14.05	2.645	18.83						

Shown are means (in percentages), standard deviations, and coefficients of variation (CVs) for each of the wild soybean traits as well as their pairwise correlations (* = *P* < 0.05 from false discovery rate tests of significance)

One-way ANOVAs showed that mean protein levels did not significantly differ between seeds originating in Japan versus those from South Korea (*P* = 0.19), but mean levels of oil and all five fatty acids did exhibit significant differences (*P* < 0.05). Inspection of the means showed that oil, palmitic and oleic acid levels were higher in seeds from South Korea compared to those from Japan whereas the reverse occurred for stearic, linoleic, and linolenic acid (Additional file 2). Differences between the two means were relatively small, ranging from 0.14% (stearic acid) to 2.06% (palmitic acid).

### Linkage disequilibrium

The results of the linkage disequilibrium estimation are shown in Additional file 3 where *r* ^*2*^ values are plotted against distances (Kb units) between SNP pairs. This figure shows that the rate of LD decay is quite rapid. The highest average *r* ^2^ value is 0.47 for SNPs separated by distances between 0 and 1 Kb, but falls to less than 0.03 for SNPs separated by about 100 Kb.

### Association mapping

**Fig. 1:**
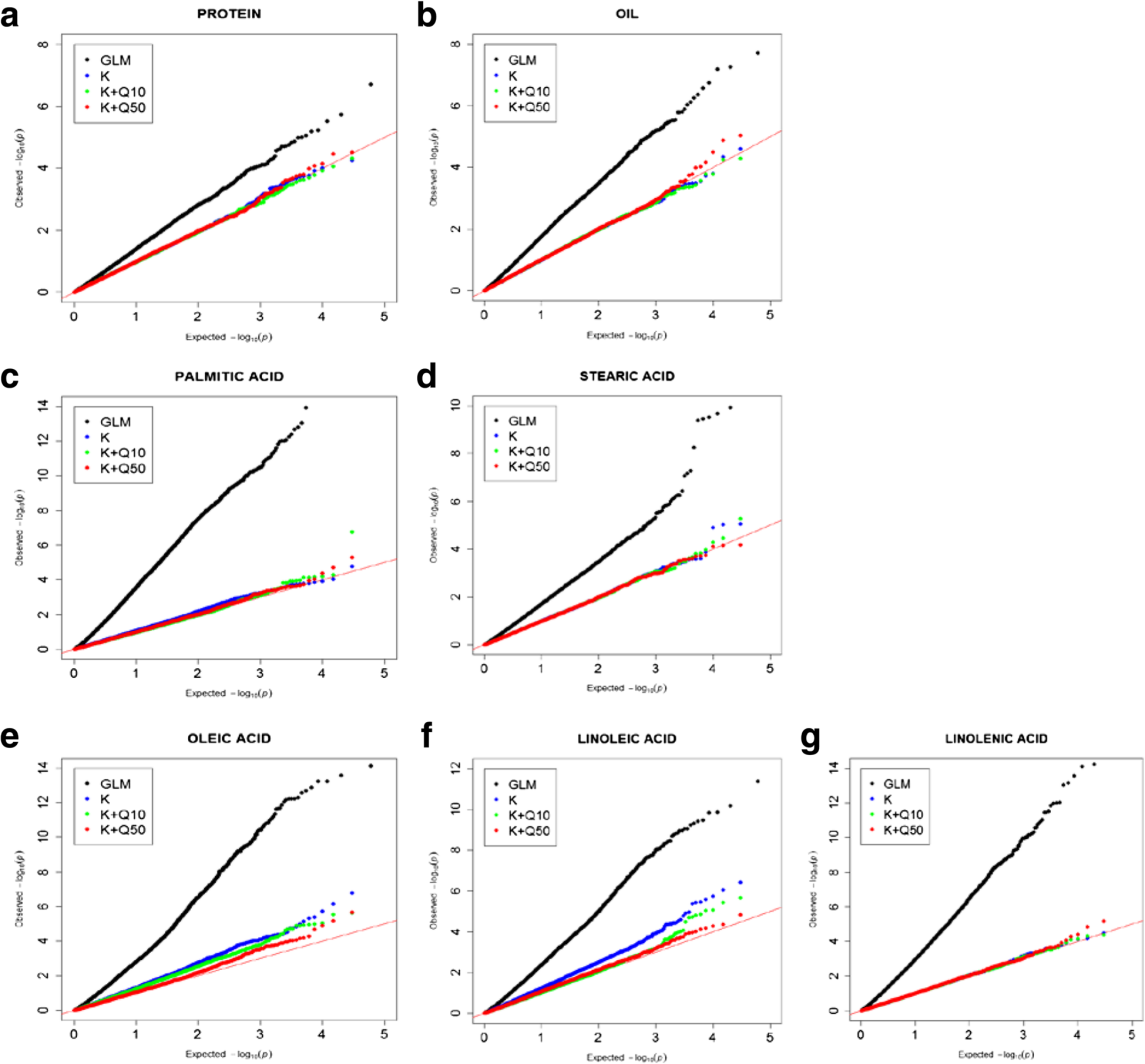
Comparison of QQ plots using different GWA models for the wild soybean seed composition traits. Shown are plots of observed and expected –log_10_ *P* values for protein **a**, oil **b**, palmitic acid **c**, stearic acid **d**, oleic acid **e**, linoleic acid **f** and linolenic acid **g**. Shown are results for the general linear model (GLM) and composite mixed linear models (CMLMs) with a kinship matrix (**K**) alone and with **K** plus the first 10 (**Q10**) and 50 (**Q50**) principal components from a principal components analysis of the SNP data

Association runs in TASSEL were performed for a number of CMLM models as previously described, and yielded QQ plots that all were an improvement over the GLM model (Fig. 1). This was especially the case for models including principal components that adjusted for population structure (a PCA bi-plot is shown in Additional file 4). Based on inspections of the QQ distributions and the calculated genomic inflation values, the **K** + **Q50** model was considered optimal for protein, oil, palmitic acid, oleic acid and linolenic acid whereas the **K** + **Q10** model appeared most appropriate for stearic acid and linoleic acid. Genomic inflation values for these models varied from 0.97 to 1.02 for all traits except oleic acid which exhibited a slightly inflated value of 1.08 even with the **K + Q50** model (Fig. 1). Using these two models, the CMLM analyses produced a total of 29 SNPs associated with the seed composition traits, with details summarized in Table 2.

**Table 2:** SNPs associated with each of the wild soybean seed composition traits

Trait	SNP	Chr	bp	*P*	- Log_10_ *P*		Allelic effect	%	h^2^
PROTEIN	ss715617976	14	24367698	3.10E-05	4.51	A	0.551	3.41	0.22
PROTEIN	ss715631057	18	45438677	3.42E-05	4.47	A	0.574	3.09	
OIL	ss715585518	3	34839398	9.21E-06	5.04	A	0.527	3.53	0.32
OIL	ss715637730	20	36705945	1.31E-05	4.88	A	0.479	3.31	
OIL	ss715637732	20	36710220	3.24E-05	4.49	A	0.422	2.99	
PALMITIC ACID	ss715595323	6	5303094	1.91E-05	4.72	A	−0.567	3.87	0.45
PALMITIC ACID	ss715597684	7	37911072	5.07E-06	5.29	A	−0.646	3.40	
PALMITIC ACID	ss715617910	14	22537916	4.15E-05	4.38	C	0.801	3.13	
STEARIC ACID	ss715618427	14	17499955	5.17E-05	4.29	A	0.704	2.95	0.50
STEARIC ACID	ss715618430	14	17561477	3.37E-05	4.47	A	0.753	3.15	
STEARIC ACID	ss715625341	16	6804502	5.31E-06	5.27	C	−0.745	3.76	
OLEIC ACID	ss715596070	7	11603310	1.25E-05	4.90	G	0.545	3.39	
OLEIC ACID	ss715617910	14	22537916	2.06E-05	4.69	C	0.693	3.13	0.36
OLEIC ACID	ss715623399	16	1258943	2.23E-06	5.65	A	−0.472	2.93	
OLEIC ACID	ss715623400	16	1260003	6.66E-06	5.18	A	−0.374	2.76	
OLEIC ACID	ss715633271	19	12874336	5.24E-05	4.28	G	−0.588	2.49	
LINOLEIC ACID	ss715582510	2	39934224	8.71E-06	5.06	A	−0.691	3.60	0.49
LINOLEIC ACID	ss715582512	2	39940256	1.57E-05	4.80	A	−0.692	3.41	
LINOLEIC ACID	ss715596075	7	11584261	2E-05	4.70	C	0.546	2.72	
LINOLEIC ACID	ss715596074	7	11585878	1.39E-05	4.86	C	−0.562	3.35	
LINOLEIC ACID	ss715596072	7	11592971	9.15E-06	5.04	C	−0.577	3.52	
LINOLEIC ACID	ss715596071	7	11602516	1.88E-05	4.73	A	−0.549	3.64	
LINOLEIC ACID	ss715596070	7	11603310	2.10E-06	5.68	G	−0.636	3.38	
LINOLEIC ACID	ss715596058	7	11756838	3.62E-06	5.44	C	0.696	4.17	
LINOLEIC ACID	ss715604488	9	44197778	3.2E-05	4.50	A	−0.430	4.04	
LINOLENIC ACID	ss715583655	2	573749	5.04E-05	4.30	C	0.745	3.72	0.44
LINOLENIC ACID	ss715583662	2	578327	1.46E-05	4.84	C	0.741	3.34	
LINOLENIC ACID	ss715617909	14	22514991	3.99E-05	4.40	C	−0.842	3.01	
LINOLENIC ACID	ss715617910	14	22537916	6.71E-06	5.17	C	−0.891	2.92	

Shown are the locations (in base pairs, bp), standardized allelic effects, and the percentage (%) of the total phenotypic variation explained for SNPs (*r* ^2^ X 100) on all chromosomes (Chr) associated with the wild soybean traits. The allelic effect is the standardized effect of the allele listed (A, C, G) compared with the alternate allele and also represents the standardized difference in the means of the two homozygous genotypes. All –log_10_ *P* scores (where *P* = the probability of SNP/trait associations) exceeding 5.348 are significant at the 5% experimentwise level and those exceeding 4.248 are significant at the chromosome-wide level. Heritabilities (h^2^) calculated using all SNPS also are given for each trait

**Fig. 2:**
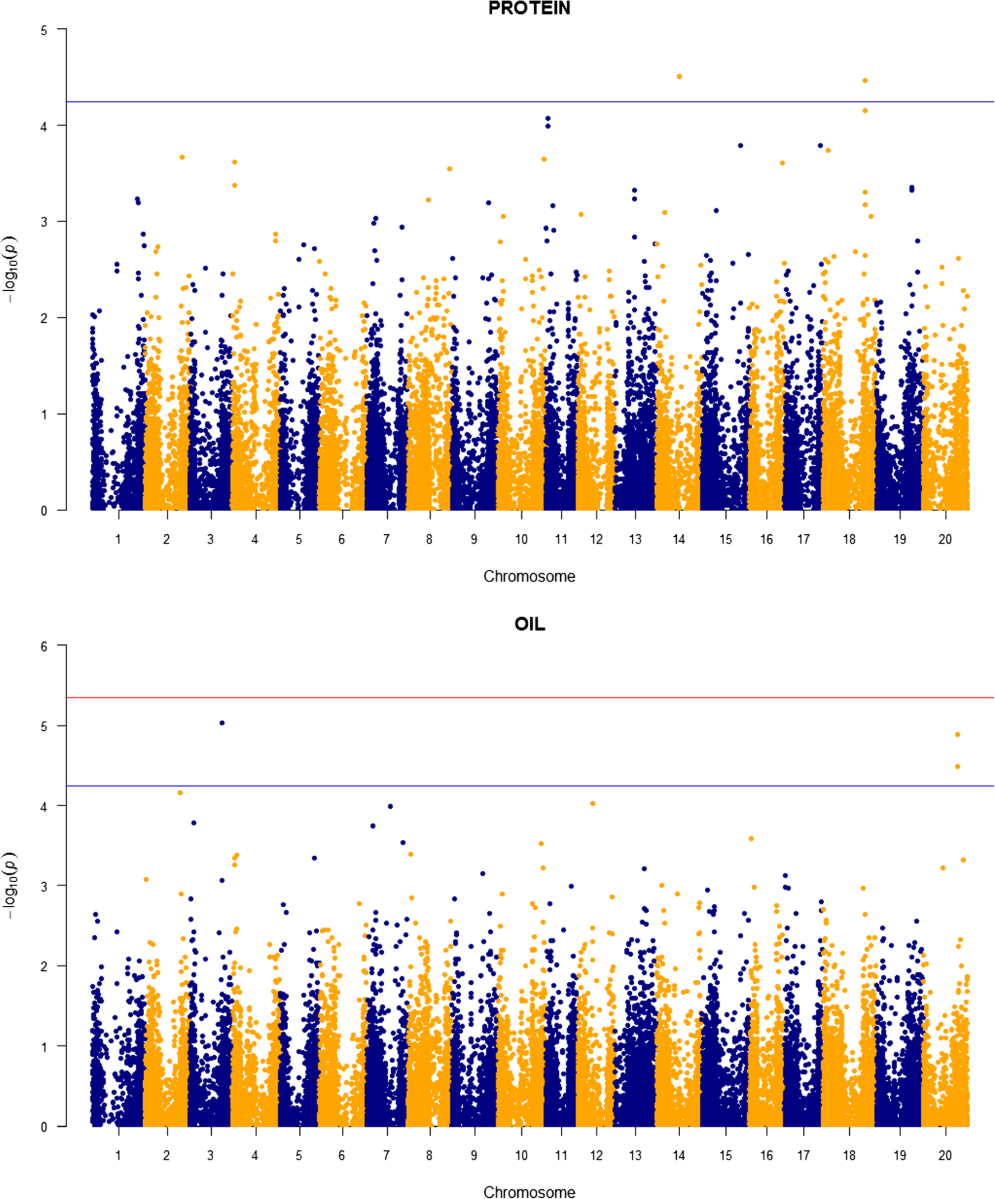
Manhattan plots for protein and oil content in the wild soybean sample. The *red horizontal line* denotes the experimentwise threshold and the *blue line* denotes the chromosome-wide threshold

For protein, two SNPs on chromosomes 14 and 18 reached the chromosome-wide threshold of association (Table 2; Fig. 2). Three SNPs were associated with oil, one on chromosome three and two close to each other on chromosome 20. All five SNPs exhibit additive effects of about ½ standard deviations and contribute 3% or more to the total phenotypic variation in these traits. The heritability estimates for protein (0.22) and oil (0.32) estimated in TASSEL were quite low (Table 2), especially for protein.

**Fig. 3:**
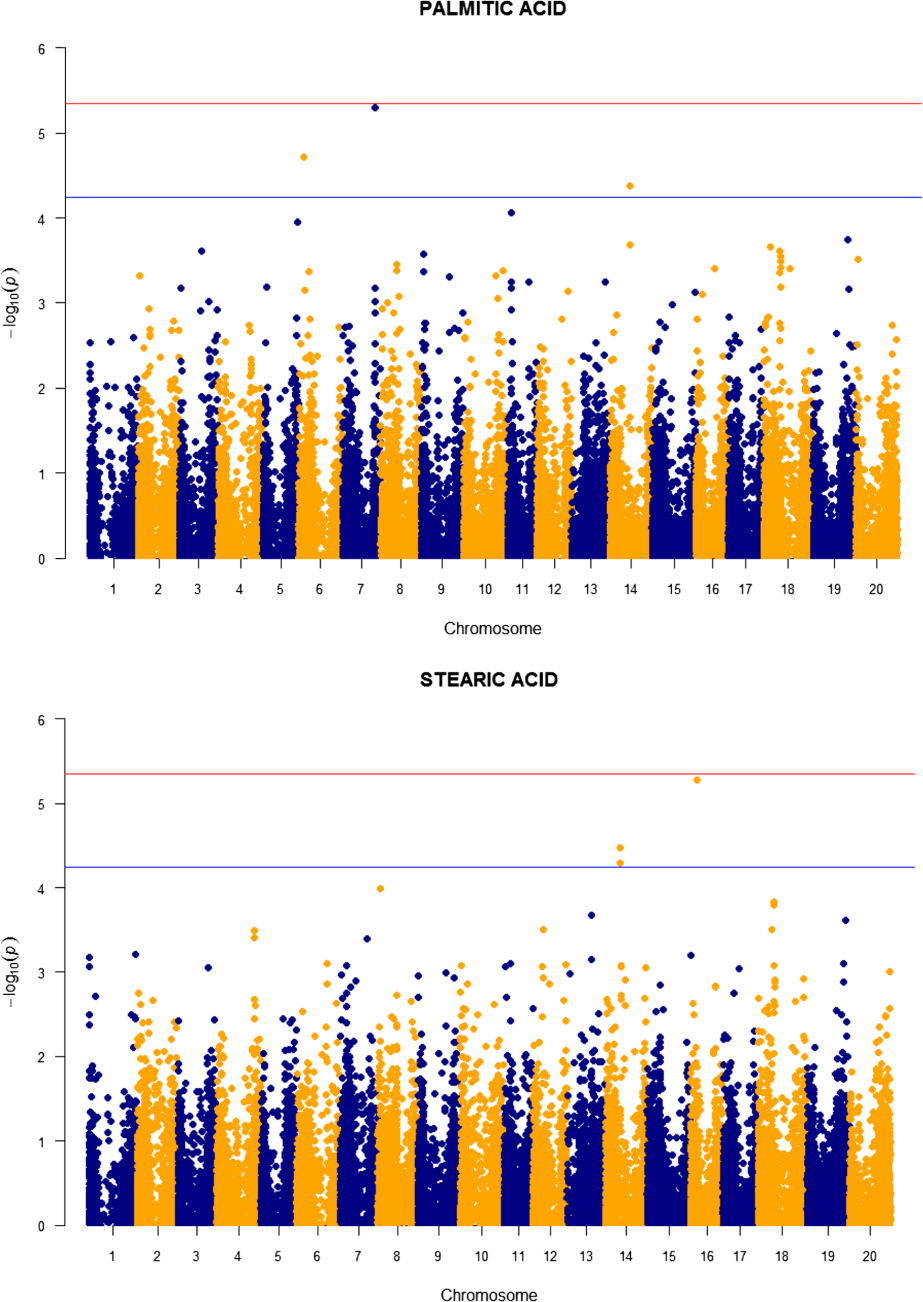
Manhattan plots for the two saturated fatty acids in the wild soybean sample. The *red horizontal line* denotes the experimentwise threshold and the *blue line* denotes the chromosome-wide threshold

**Fig. 4:**
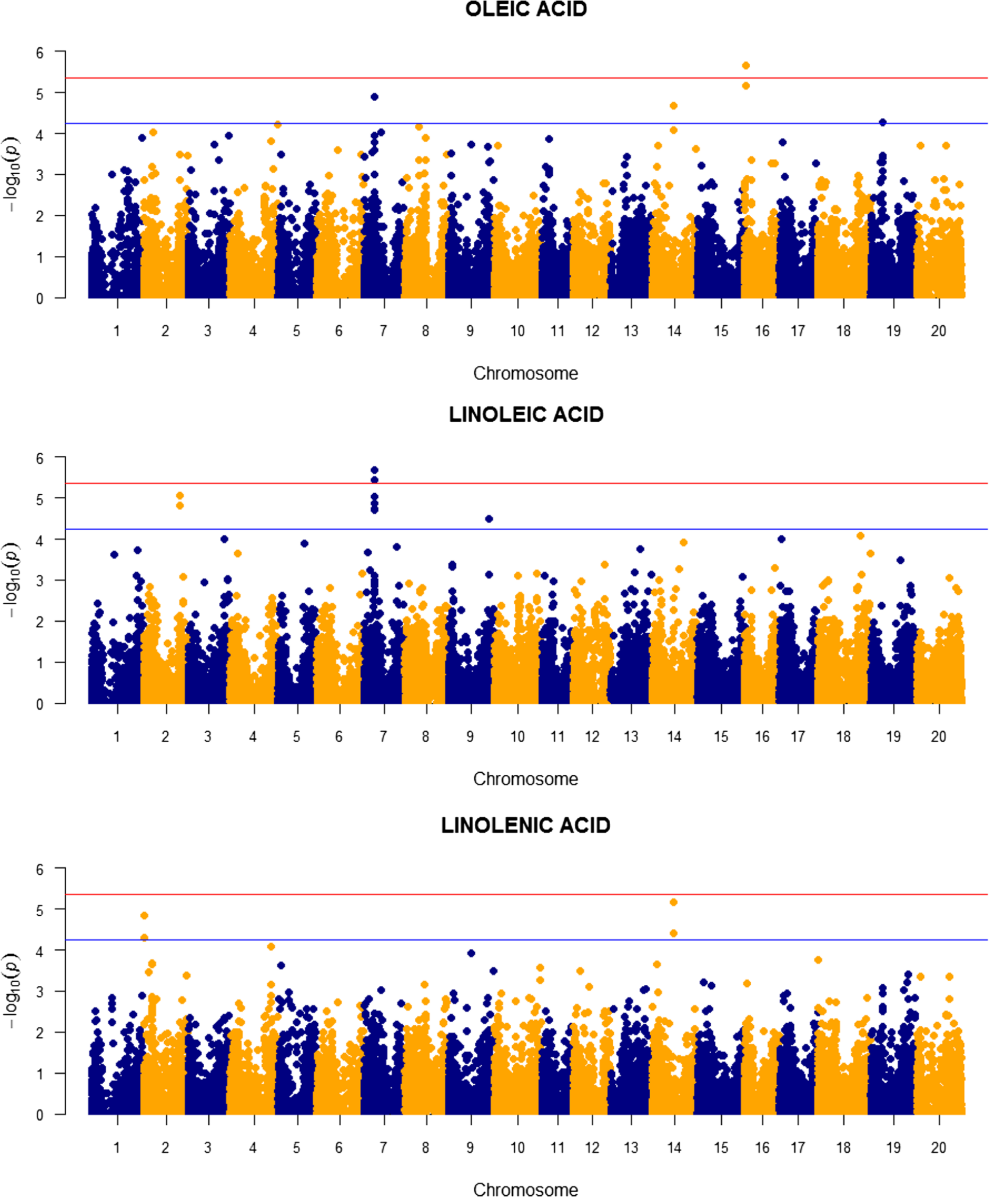
Manhattan plots for the three unsaturated fatty acids in the wild soybean sample. The *red horizontal line* denotes the experimentwise threshold and the *blue line* denotes the chromosome-wide threshold

TASSEL runs uncovered a total of 24 SNPs associated with the fatty acids, including one on chromosome 16 for oleic acid and two on chromosome seven for linoleic acid that reached the 5% genome-wide significance level (Table 2, Figs. 3 and 4). These SNPs are located on seven different chromosomes, with three on chromosome 16, four on chromosome two, six on chromosome 14, and eight on chromosome seven. The fourteen SNPs located on chromosomes seven and 14 collectively are the most prominent feature in the Manhattan plots (Figs. 3 and 4). The SNPs contribute on average 3.32% of the total variation in the fatty acids. Heritabilities estimated for these traits generally are higher than those for protein and oil, ranging from 0.44 to 0.49 and averaging 0.45.

Three SNPs were associated with each of the two saturated fatty acids, palmitic acid and stearic acid (Table 2, Fig. 3). Two stearic acid SNPs are clustered together on chromosome 14 (17.50—17.56 Mb), but are at a different location than the chromosome 14 SNP associated with palmitic acid (22.5 Mb) and another SNP on this chromosome associated with protein (24.4 Mb). Allelic effects for the underlying QTLs affecting palmitic and stearic acid tend to be higher than for those affecting protein and oil.

For the unsaturated fatty acids, five SNPs were associated with oleic acid, 9 with linoleic acid, and four with linolenic acid (Table 2, Fig. 4) A chromosome 14 SNP at 22537916 bp is associated with both oleic and linolenic acid, as well as palmitic acid, suggesting pleiotropy of an underlying gene that is affecting all three traits. Pleiotropy also is apparent with a SNP on chromosome seven at 11603310 bp associated with both oleic and linoleic acid. All other SNPs, however, appear to occupy unique chromosomal locations. Allelic effects of SNPs associated with the unsaturated fatty acids are highest for linolenic acid, ranging from 0.75 to 0.89 standard deviations.

### QTLs and candidate genes

A total of 29 SNPs are significantly associated with the seed composition traits in our wild soybean populations. Eight co-located with QTLs previously identified in cultivated soybean populations (Table 3), including two each for oil and stearic acid, one for linoleic acid, and one for linolenic acid. We also found a number of genes that harbor, or are adjacent to, the 29 SNPs associated with the seed composition traits (Additional file 5). Some of these genes are involved in fatty acid metabolism and regulations, such as those encoding plant stearoyl-acyl-carrier-protein desaturase family protein, and phospholipase D alpha 1. (Additional file 5).

**Table 3:** Soybean QTLs and candidate genes for the peak SNPs found for the 7 soybean traits

TRAIT	SNP	CHR	BP	Previous QTLs	Gene ID
PROTEIN	ss715617976	14	24367698		
PROTEIN	ss715631057	18	45438677	[57]	
OIL	ss715585518	3	34839398		
OIL	ss715637730	20	36705945	[58]	Glyma.20G124700.1
OIL	ss715637732	20	36710220	[58]	Glyma.20G124700.1
PALMITIC ACID	ss715595323	6	5303094		
PALMITIC ACID	ss715597684	7	37911072		
PALMITIC ACID	ss715617910	14	22537916		
STEARIC ACID	ss715618427	14	17499955	[59]	Glyma.14G121400.1
STEARIC ACID	ss715618430	14	17561477	[59]	
STEARIC ACID	ss715625341	16	6804502		Glyma.16G068500.1
OLEIC ACID	ss715596070	7	11603310	[60]	
OLEIC ACID	ss715617910	14	22537916		
OLEIC ACID	ss715623399	16	1258943		
OLEIC ACID	ss715623400	16	1260003		
OLEIC ACID	ss715633271	19	12874336		
LINOLEIC ACID	ss715582510	2	39934224		
LINOLEIC ACID	ss715582512	2	39940256		
LINOLEIC ACID	ss715596075	7	11584261		Glyma.07G112100.1
LINOLEIC ACID	ss715596074	7	11585878		Glyma.07G112100.1
LINOLEIC ACID	ss715596072	7	11592971		
LINOLEIC ACID	ss715596071	7	11602516		
LINOLEIC ACID	ss715596070	7	11603310		
LINOLEIC ACID	ss715596058	7	11756838		
LINOLEIC ACID	ss715604488	9	44197778		Glyma.09G218700.1
LINOLENIC ACID	ss715583655	2	573749		
LINOLENIC ACID	ss715583662	2	578327		Glytma.02G005200.1
LINOLENIC ACID	ss715617909	14	22514991	[60]	
LINOLENIC ACID	ss715617910	14	22537916	[60]	

Gene IDs are given for soybean genes containing the SNPs and are based on SoyBase searches

## Discussion

The intent of this investigation was to identify SNPs and candidate genes that play an important role in the seed composition trait variation in our wild soybean population. We were especially interested to see whether we might identify a number of novel QTLs not discovered in the linkage or association mapping studies previously done with cultivated soybean samples. By using GWA strategy with high-density of genome-wide SNPs, we were able to uncover SNP associations for these traits, some of which co-localized with previously-identified QTLs in cultivated soybean populations whereas others appeared to be novel. Below we discuss details regarding these SNPs and their potential underlying candidate genes affecting each of the traits.

### Wild soybean protein and oil

The protein content in our wild soybean sample averaged about 48%, a higher mean percentage than typically is seen in cultivated soybeans [6, 17]. Consistent with this, Chen and Nelson [33] found that the mean protein level in a wild soybean sample was significantly higher than that in a sample of cultivated soybeans. The conventional explanation for this disparity is that selection for cultivated soybean varieties generally has focused on increased yield and oil content [19], traits that tend to be negatively related to protein content [34, 35, 36]. Estimates of the phenotypic correlation of protein and oil content in cultivated soybeans range from −0.62 to −0.78 [7, 20, 21], and some loci affecting both traits exhibit antagonistic pleiotropy [20]. Our estimated protein/oil correlation of −0.36 (Table 1) was considerably lower, and presumably is a reflection of reduced selection pressure on oil content in wild soybeans.

The genetic variability in protein content as estimated by its heritability was a rather low 0.22 and thus it is not surprising that only two SNPs associated with this trait reached even the suggestive threshold level. This heritability estimate for protein is strictly applicable to our specific sample of (maturity groups V through IX) wild soybean accessions and thus estimates from other studies may be very different depending upon the number of QTLs segregating for protein, the maturity groups sampled, and various other factors. For example, the heritability of protein content estimated by Hwang et al. [7] in a GWA study of cultivated soybeans was a quite high 0.78. Consistent with this, Hwang et al. [7] also uncovered 40 SNPs significantly associated with protein content, although used a very low threshold of −log_10_ *P* = 3.0. Only five of their 40 SNPs, all tightly clustered on chromosome 20 [7] exceeded our -log_10_ *P* suggestive threshold of 4.248. On the other hand, Bandillo et al. [20] used a more conservative -log_10_ *P* threshold of 5.17, and found 19 SNPs (11 clustered on chromosome 20) associated with protein in their domesticated soybean sample.

The oil content in the wild soybeans we analyzed averaged 11%, well below the 15–25% levels typically found in cultivated soybeans [18]. Selection for increased oil content has been practiced for many years, and Zhou et al. [19] recently have identified 96 separate selective sweeps located within known oil QTL regions. Oil variability in our wild soybean sample as assessed by the coefficient of variation (10.8) was higher than comparable estimates for cultivated soybeans such as that of 4.74 calculated by Akond et al. [6] among recombinant inbred lines.

As was the case for protein content, the heritability for oil content was considerably lower (0.32) than various estimates such as 0.66 [17] and 0.78 [7] made for cultivated soybean samples. These consistently higher levels of heritability for both protein and oil content in soybean may well reflect the past history of selection for these traits among a number of different soybean populations. Three SNPs in the wild soybeans showed associations with oil, including one on chromosome three in a region not enclosing any previously-discovered QTLs or genes affecting oil (Table 3). This makes the identity of the candidate gene underlying this association more speculative, although some possibilities are listed in Additional file 5. Two other SNPs on chromosome 20 are located within Glyma.20G124700.1, which therefore is a strong possibility for a candidate gene affecting protein.

### Wild soybean saturated fatty acids

The mean for palmitic acid in our wild soybean sample was nearly 13%, slightly higher than the 11% [37] to 12% [5] levels typically found in soybean oil. Because this predominant saturated fatty acid in cultivated soybeans is associated with cardiovascular problems [38], efforts have been made to reduce its level [5, 39, 40]. Considerable success has been achieved with the discovery of alleles at two independent loci*, fap1* on chromosome nine and *fap3* on chromosome five, either of which can reduce palmitic acid levels to as low as 6% or even lower [41, 42]. Unfortunately, these alleles also tend to decrease overall yield [42] and thus compromise their usefulness in soybean breeding regimes. This suggests that a useful alternate might be to screen wild soybeans for accessions with reduced palmitic acid levels to identify novel genes for eventual transfer to domesticated varieties. The palmitic acid level in our accessions from Japan was less than for those from South Korea when all plants were grown in a common environment, so there clearly is some natural genetic variation for this trait in wild soybeans across different geographic areas.

Beyond major gene effects, many QTLs with minor effects on palmitic acid levels have been discovered in soybean [5, 18], and our study uncovered three additional QTLs for this trait in wild soybeans that all appear to be in novel positions. The SNP on chromosome 14 at 22537916 bp had the greatest effect, with the TT genotype at this marker decreasing the palmitic acid mean from 12.95% in the CC genotype to 12.15%. Selection for the T allele at this marker therefore might be useful in reducing the palmitic acid level, but this same allele also reduced oleic acid (Table 2) from about 15% in the GG genotype to 12% in the TT genotype). Although this is consistent with the positive correlation between palmitic and oleic acid in our sample (Table 1), increases rather than decreases in oleic acid are considered optimal for cardiovascular health [3]. A better strategy therefore would be to select for QTLs affecting palmitic acid, such as those we found on chromosomes six and seven (Table 2), that have no pleiotropic effects on oleic acid.

We also discovered three SNPs associated with the other saturated fatty acid, stearic acid (Table 2). Two were clustered on chromosome 14, and it seems quite likely that the candidate gene underlying both SNPs is Glyma.14G121400.1 that codes for plant stearoyl-acyl-carrier-protein desaturase. This is an enzyme that catalyzes the conversion of stearoyl-ACP to oleoyol-ACP and plays an important role in the biosynthesis of unsaturated fatty acids (specifically, oleic acid) from saturated fatty acids [43]. The other SNP associated with stearic acid is located in the soybean gene Glyma.16G068500.1 that codes for surfeit locus protein two (SURF2). Ma et al. [44] found that this protein was differentially expressed after 6 and 12 h of rehydration of desiccated *Myrothamnus flabellifolia* branches, with the fatty acid biosynthetic pathway among those significantly enriched during the rehydration process.

### Wild soybean unsaturated fatty acids

Oleic acid is a monounsaturated fatty acid that at high levels is associated with increased health benefits as well as oxidative stability [5, 45]. In soybean, this fatty acid typically averages 20–25% [46, 47], although its level in our wild soybean sample was considerably lower than this (overall average = 15%), especially in accessions from Japan. This difference might partially be explained by selection for overall oil content in domesticated soybeans that has increased oleic acid levels as well. Consistent with this, oleic acid in our wild soybean sample was the only one of the five fatty acids to exhibit a significant, positive (although low) correlation with total oil content (Table 1). Direct selection for increased oleic acid itself also has been practiced in domesticated soybeans, especially with the discovery of mutant *FAD2* genes that can increase its level to as much as 80% or more [47, 48]. This approach appears very promising, although in some populations these mutant alleles may negatively impact yield [5].

We found five SNPs associated with oleic acid, including one on chromosome 14 in the same position as a SNP associated with palmitic acid. Two SNPs clustered on chromosome 16 generated the highest –log_10_ *P* values, but mapped in a region where no previous QTLs affecting oleic acid have been reported. Among the candidate gene possibilities in this region is Glyma.16G014000 that codes for the enzyme pectin methylesterase 1 (Additional file 5) that in kiwis is influenced by the level of oleic acid [49]. An oleic acid SNP on chromosome seven was interesting because it colocalized with another SNP associated with linoleic acid, discussed below. A final SNP on chromosome nine showed an association with oleic acid, but it barely reached the suggestive threshold and in fact would fall below this level with an appropriate adjustment for the genomic inflation in this trait.

Linoleic acid is a polyunsaturated fatty acid that, unlike oleic acid, does not exhibit oxidative stability and therefore can quickly become rancid, effectively reducing its shelf life [10, 47]. To remedy this, soy oil typically is hydrogenated, but this produces *trans*-fats that are associated with cardiovascular problems [4, 50]. Linoleic acid makes up the largest proportion (typically about 2%) in soy oil (Fehr [37]), as it also did in our wild soybean sample (mean = 54%), so there has been a considerable incentive to considerably reduce the level of this fatty acid. Fortunately, the mutant *FAD2* genes discussed above do precisely this by largely disrupting the conversion of oleic acid precursors into linoleic acid precursors [47]. As a consequence, less emphasis appears to have been put on discovering major genes that reduce linoleic acid levels, although a number of QTLs with minor effects on fatty acid have been discovered [6, 18].

Our CMLM analysis produced nine SNPs associated with linoleic acid, the highest number for any of the seven traits. Most notable were six SNPs clustered in an interval from 11.58 to 11.75 Mb on chromosome seven, an area where no previous QTLs affecting this trait have been found. Two SNPs in the proximal part of this region (at ll.58 Mb) are found within Glyma.07G112100.1, and three other SNPs (11.59 to 11.60 Mb) are within 50 Kb of this gene. In *Arabidopsis*, this gene codes for a mitochondrial transcription termination factor (MTERF) family protein. Babiychuk et al. [51] characterized a number of *Arabidopsis* proteins in the MTERF family, and showed that they are essential for plastid gene expression and plant development, including biosynthesis of fatty acids. Thus this gene would appear to be a strong candidate for the QTLs on chromosome seven affecting linoleic acid in the wild soybeans. Glyma.07G112100.1 also is within 50 Kb of the SNP on chromosome seven associated with palmitic acid (Additional file 5), so seems a likely candidate gene for that fatty acid as well.

Like linoleic acid, linolenic acid also is a polyunsaturated fatty acid although its proportion in soy oil is much less, typically about 8–10% [52]. Three key loci coding for omega-3 fatty acid desaturases (FAD3A, FAD3B, and FAD3C) have been discovered that convert linoleic acid into linolenic acid [47, 48]. Further, marker assays now have been developed to allow breeders to screen for mutants at these loci that decrease linoleic acid to desirable levels [47]. As was true for the other fatty acids, however, a number of other QTLs affecting linoleic acid have been discovered [10, 18]. We found four SNPs associated with linoleic acid, including one on chromosome 14 at precisely the same location (22537916 bp) as others affecting both palmitic and oleic acid. Clearly there appears to be a candidate gene in this area that is pleiotropically affecting three of the five fatty acids.

### QTL and candidate gene considerations

We discovered a total of 29 SNPs on ten different chromosomes that were associated with the seed composition traits in our wild soybean sample. SNPs in clusters, especially those on chromosomes seven and 14, probably are of most interest and worth further investigation, but all of the significant SNPs affecting these traits would need to be verified in subsequent studies. If some of these SNPs/candidate genes and their effects on the seed composition traits are validated in future studies, this should provide valuable information about the genetic basis of protein and oil biosynthesis in wild soybean. They may also prove worthwhile for eventual introgression into soybean lines to enhance breeding efforts for increased protein or oil content and/or suggest additional genetic control of pathways involved in seed composition biosynthesis.

Eight of the 29 significant SNPs co-localized with previously-identified QTLs in cultivated soybean populations and some of the candidate genes identified here are involved in fatty acid metabolism and regulations. It was not surprising, however, that some of the well-studied fatty acid pathway genes were not identified in our wild soybean sample. There are several potential reasons for this: 1) The genetic architecture of most quantitative traits is very complex and population-specific, with different QTLs/candidate genes for the same trait typically identified in different populations and/or species. For example, Li et al. [18] used 1205 SNPs developed for more than 600 candidate genes identified in the model plant *Arabidopsis*, and found that only a small fraction of these SNPs (37 out of 1205) showed significant associations with fatty acid biosynthetic genes in soybean. The wild soybean used here is the closest wild relative of cultivated soybean, but has a substantially higher level of genetic diversity. We therefore did not expect to find the same SNPs/genes controlling the seed composition traits in our sample as those previously discovered in the more intensively-studied cultivated soybean populations. 2) This result may simply be a reflection of the fact that these loci were not polymorphic in our sample. Or even if some of these genes turned out to be polymorphic, they could have been missed because of a lack of sufficiently close SNP markers. This is especially the case because the linkage disequilibrium blocks in wild soybean populations are well known to be much less extensive than those in domesticated soybean samples. We investigated these possibilities for the well-studied genes, *fap1* (chromosome nine) and *fap3* (chromosome five), in our genomic data, and found that the closest SNPs were within 58.6 (*fap1*) and 606.8 kb (*fap3*) of the locations of these genes. For *fap3* especially, therefore, this suggests that we may not have had adequate coverage of SNP markers to detect the effect of this gene, if segregating. 3) Some loci previously discovered as affecting these traits might also have been missed if SNPs were eliminated that did not meet the filtering criteria. 4) Given that most genes exhibit interactions (epistasis), some genes known to affect the seed composition traits may have had weaker signals in wild soybean population than in other cultivated soybean populations, and their effects may not have met the stringent statistical threshold for detection.

Regardless of the actual number of QTLs affecting the seed composition traits in our wild soybean sample, SNP variation for each of the traits was not nearly enough to account for their total genetic variance as estimated by their heritabilities. This ‘missing heritability’ is common in GWA studies and is generally ascribable to several factors such as incomplete linkage between the underlying genes and the closest markers and/or the presence of rare variants that affect the traits of interest [12, 15, 53]. The QTL results for the traits in our sample of wild soybeans presumably reflect these and various other factors. One factor may have been LD which in the wild soybean genome spans much shorter distances than in soybean, suggesting that the use of many more markers would have resulted in the detection of additional QTLs. Among the available soybean SNPs, we filtered those with minor frequencies less than 0.05, some of which may have been linked to genes affecting the traits. If so, an alternative strategy would be to use a linkage mapping approach where it is possible to construct crosses that would produce an F_2_ generation with minor alleles at moderate frequencies. We also were quite successful in adjusting for population structure and thus reducing genomic inflation that would have resulted in higher -log_10_ *P* association scores and thus more SNPs associating with the wild soybean traits.

Beyond these kinds of considerations, it may well be that the seed composition traits in our wild soybean sample are affected by a number of genes, each with a small effect. This sort of genetic architecture is common in a number of traits, but detection of relatively weak signals presents special difficulties for the GWA approach [15, 53]. As a potential example of this, the SNP on chromosome 14 (at 22537916 bp) affecting palmitic acid, oleic acid, and linolenic acid also exhibited association scores of 2.26 (*P* = 0.005) with linoleic acid and 2.91 (*P* = 0.0013) with stearic acid. Although these scores are well below the suggestive threshold, it is possible that the underlying QTL in this region pleiotropically affects all five rather than three fatty acids but we simply do not have sufficient power to detect its effect on linoleic and stearic acid.

The QTLs controlling variation in the seed composition traits in wild soybeans also may exhibit interactions within loci (dominance) or between loci (epistasis). We ran a preliminary analysis that included heterozygous SNPs and uncovered suggestive evidence of dominance effects, but the sample sizes for the heterozygotes in most cases were so small (some were as low as 1) that these results appeared unreliable and could represent false positives [54]. The linkage mapping approach generally would seem to be a better strategy for the detection of dominance effects in generally selfing organisms such as *G. soja*. Non-additive epistatic effects among different loci also may be an important part of the genetic architecture of these traits. Tests for epistasis in GWA studies are technically difficult [55] and only rarely have been attempted [56]. As computer and statistical techniques for the detection of epistasis in these studies evolve, we predict that these effects will explain some of the hidden genetic variability in many traits, including those we have analyzed in wild soybeans.

## Conclusions

This GWA study is the first conducted on seed composition traits measured solely in a wild soybean population, and revealed a number of QTLs that have not been previously discovered. Some of these QTLs may be useful to breeders who select for increased protein/oil content or altered fatty acid ratios in soybean seeds. Our results also provide additional insight into the genetic architecture of these traits in a large sample of wild soybean, and suggest some new candidate genes whose molecular effects on these traits need to be further studied.

## Additional files

Additional file 1: The country and province where the 570 accessions (PI) used in the analysis originated. Blank entries indicate unknown origins. (DOCX 37 kb)
Additional file 2: Plots of the mean values of the 7 soybean traits from accessions sampled from Japan versus South Korea. (DOCX 15 kb)
Additional file 3: Genome-wide average LD decay estimated for the wild soybean sample. The LD decay is measured in r^2^ values between SNPs as a function of the distance between them. (DOCX 35 kb)
Additional file 4: A PCA bi-plot showing the genetic population structure of the studied accessions. (DOCX 379 kb)
Additional file 5: Candidate genes for the top SNPs associated with the seed composition traits. Soybean genes located in regions 50 kb in both directions from top SNPS. Shown are the locations of each SNP, the soybean gene ID and the *Arabadopsis* best hit, symbol and description. (XLSX 46 kb)

## References

[1] Wilson RF, Stacey G (1988). Soybean: market driven research needs. Genetics and genomics of soybean.

[2] Willett WC (1994). Diet and health: what should we eat?. Science.

[3] Beare-Rogers J, Przybylski R, McDonald BS (1995). Food fats and FA in human nutrition. Vegetable oils for human nutrition.

[4] Mozaffarian D, Katan MB, Ascherio A, Stampfer MJ, Willett WC (2006). Trans fatty acids and cardiovascular disease. N Engl J Med.

[5] Lee J-D, Bilycu KD, Shannon JG (2007). Genetics and breeding for modified fatty acid profile in soybean seed oil. J Crop Sci Biotech.

[6] Akond M, Liu S, Boney M, Kantartzi SK, Meksem K, Bellaloui N, Lightfoot DA, Kassem MA (2014). Identification of quantitative trait loci (QTL) underlying protein, oil, and five major fatty acids’ contents in soybean. Amer J Plant Sci.

[7] Hwang E-Y, Song Q, Jia G, Specht JE, Hyten DL, Costa J, Cregan PB (2014). A genome-wide association study of seed protein and oil content in soybean. BMC Genomics.

[8] Hyten DL, Pantalone VR, Saxton AM, Schmidt ME, Sams CE (2004). Molecular mapping and identification of soybean fatty acid modifier quantitative trait loci. J Amer Oil Chem Soc.

[9] Panthee DR, Pantalone VR, Saxton AM (2006). Modifier QTL for fatty acid composition in soybean oil. Euphytica.

[10] Wang X, Jiang G-L, Green M, Scott RA, Hyten DL (2014). Quantitative trait locus analysis of unsaturated fatty acids in a recombinant inbred population of soybean. Mol Breeding.

[11] Borevitz JO, Nordborg M (2003). The impact of genomics on the study of natural variation in *Arabidopsis*. Plant Physiol.

[12] Ingvarsson PK, Street NR (2011). Association genetics of complex traits in plants. New Phytol.

[13] Chaudhary J, Patil GB, Sonah H, Deshmukh RK, Vuong TD, Valliyodlan B, Nguyen HT (2015). Expanding omics resources for improvement of soybean seed composition traits. Front Plant Sci.

[14] Astle W, Balding DJ (2009). Population structure and cryptic relatedness in genetic association studies. Stat Sci.

[15] Korte A, Farlow A (2013). The advantages and limitations of trait analysis with GWAS: a review. Plant Methods.

[16] Sillanpaa MJ (2011). Overview of techniques to account for confounding due to population stratification and cryptic relatedness in genomic data association analyses. Heredity.

[17] Vaughn JN, Nelson RL, Song Q, Cregan PB, Li Z (2014). The genetic architecture of seed composition in soybean is refined by genome-wide association scans across multiple populations. G3: Genes|Genomes|Genetics.

[18] Li Y-H, Reif JC, Ma Y-S, Hong H-L, Liu Z-X, Chang R-Z, Qiu L-J (2015). Targeted association mapping demonstrating the complex molecular genetics of fatty acid formation in soybean. BMC Genomics.

[19] Zhou Z, Jiang Y, Wang Z, Gou Z, Lyu J, Li W, Yu Y, Shu L, Shao Y, Ma Y, Fang C, Shien Y, Liu T, Li C, Li Q, Wu M, Wang M, Wu Y, Dong Y, Wan W, Wang X, Ding Z, Gao Y, Xiang H, Zhu B, Lee S-H, Wang W, Tian Z (2015). Resequencing 302 wild and cultivated accessions identifies genes related to domestication and improvement in soybean. Nature Biotech.

[20] Bandillo N, Jarquin D, Song Q, Nelson R, Cregan P, Specht J, Lorenz A (2014). A population structure and genome-wide association analysis on the USDA soybean germplasm collection. Plant Genome.

[21] Sonah H, O’Donoughue L, Cober E, Rajcan I, Belzile F (2015). Identification of loci governing eight agronomic traits using a GBS-GWAS approach and validation by QTL mapping in soya bean. Plant Biotech J.

[22] Li YH, Li W, Zhang C, Yang L, Chang R-Z, Gaut BS, Qiu L-J (2010). Genetic diversity in cultivated soybean (Glycine max) and its wild progenitor (Glycine soja) for simple sequence repeat and single-nucleotide polymorphism loci. New Phytol.

[23] Li YH, Zhao SC, Ma JX, Li D, Yan L, Li J, Qi XT, Guo XS, Zhang L, He WM, Chang RZ, Liang QS, Guo Y, Ye C, Wang XB, Tao Y, Guan RX, Wang JY, Liu YL, Jin LG, Zhang XQ, Liu ZX, Zhang LJ, Chen J, Wang KJ, Nielsen R, Li RQ, Chen PY, Li WB, Reif JC, Purugganan M, Wang J, Zhang MC, Wang JW, Qiu LJ (2013). Molecular footprints of domestication and improvement in soybean revealed by whole genome re-sequencing. BMC Genomic.

[24] Li YH, Zhou G, Ma J, Jiang W, Jin LG, Zhang Z, Guo Y, Zhang J, Sui Y, Zheng L, Zhang SS, Xuo Q, Shi XH, Li YF, Zhang WK, Hu Y, Kong G, Hong HL, Tan B, Song J, Liu ZX, Wang Y, Ruan H, Yeung CK, Liu J, Wang H, Zhang L, Guan RX, Wang KJ, Li WB, Chen SY, Chang RZ, Jiang Z, Jackson SA, Li R, Qiu LJ (2014). De novo assembly of soybean wild relatives for pan-genome analysis of diversity and agronomic traits. Nat Biotect.

[25] Song Q, Hyten DL, Jia G, Quigley CV, Fickus EW, Nelson R, Cregan BP (2013). Development and evaluation of SoySNP50K, a high-density genotyping array for soybean. PLoS One.

[26] Kisha TJ, Sneller CH, Diers BW (1997). Relationship between genetic distance among parents and genetic variance in populations of soybean. Crop Sci.

[27] Song Q, Hyten DL, Jia G, Quigley CV, Fickus EW (2015). Fingerprinting soybean germplasm and its utility in genomic research. G3 (Bethesda).

[28] Bradbury PJ, Zhang Z, Kroon DE, Casstevens TM, Ramdoss Y, Buckler ES. TASSEL: Software for association mapping of complex traits in diverse samples. Bioinform. 2007;23:2633–5. 10.1093/bioinformatics/btm308 17586829

[29] Goh L, Yap VB (2009). Effects of normalization on quantitative traits in association test. BMC Bioinform.

[30] Benjamini Y, Hochberg Y (1995). Controlling the false discovery rate: a practical and powerful approach to multiple testing. J Roy Stat Soc B.

[31] Li J, Ji L (2005). Adjusting multiple testing in multilocus analyses using the eigenvalues of a correlation matrix. Heredity.

[32] Lander E, Kruglyak L (1995). Genetic dissection of complex traits: guidelines for interpreting and reporting linkage results. Nat Genet.

[33] Chen YW, Nelson RL (2004). Genetic variation and relationships among cultivated, wild, and semiwild soybean. Crop Sci.

[34] Helms TC, Orf JH. Protein oil, and yield of soybean lines selected for increased protein. Crop Sci. 1998;38:707–11.

[35] Li H, Barton JW (2002). Selecting increased seed density to increase indirectly soybean seed protein concentration. Crop Sci.

[36] Dong YS, Zhuang BC, Zhao LM, Sun H, He MY (2001). The genetic diversity of annual wild soybeans grown in China. Theoret Appl Genet.

[37] Fehr WR (2007). Breeding for modified fatty acid composition in soybean. Crop Sci.

[38] Hu FB, Stampfer MJH, Manson JE, Rimm E, Colditz GA, Rosner BA, Hennekens CH, Willet WC (1997). Dietary fat intake and the risk of coronary heart disease in women. New Engl J Med.

[39] Takagi Y, Rahman SM, Joo H, Kawakita T (1995). Reduced and elevated palmitic acid mutants in soybean developed by X-ray irradiation. Biosci biotech biochem.

[40] Thapa R, Carrero-Colon M, Hudson KA (2015). New alleles of FATB1A to reduce palmitic acid levels in soybean. Crop Sci.

[41] Wilson RF, Marquardt TC, Novitzky WP, Burton JW, Wilcox JR, Kinney AJ, Dewey RE (2001). Metabolic mechanisms associated with alleles governing the 16.0 concentration of soybean oil. J Amer Oil Chem Soc.

[42] Cardinal AJ, Whetten R, Wang S, Auclair J, Hyten DL (2014). Mapping the low palmitate fap1 mutation and validation of its effects in soybean oil and agronomic traits in three soybean populations. Theoret Appl Genet.

[43] Zhang Y, Maximova SN, Guiltinan MJ (2015). Characterization of a steraoyl-acyl protein desaturase family from chocolate tree, Theobroma cacao L. Front Plant Sci.

[44] Ma C, Wang H, Macnish AJ, Estrada-Melo AC, Linz J, Chang Y, Reid MS, Jiang C-Z (2015). Transcriptomic analysis reveals numerous diverse protein kinases and transcription factors involved in desiccation tolerance in the resurrection plant *Myrothamnus flabellifolia*. Horticult Res.

[45] Teres S, Barcelo-Coblijn G, Benet M, Alvarez R, Ressani R, Halver JE, Escriba PV (2008). Oleic acid content is responsible for the reduction in blood pressure induced by olive oil. Proc Nat. Acad Sci..

[46] Pantalone V, Walker D, Dewey R, Rajcan I, Wilson R, Stalker HT, Brummer EC (2004). DNA marker-assisted selection for improvement of soybean oil concentration & quality. Legume crop genomics.

[47] Shi Z, Bachleda N, Pham AT, Bilyeu K, Shannon G, Nguyen H, Li Z (2015). High-throughput and functional SNP detection assays for oleic and linolenic acids in soybean. Mol Breed.

[48] Pham AT, Shannon JG, Bilyeu KD (2012). Combinations of mutant *FAD2* and *FAD3* genes to produce high oleic acid and low linolenic acid soybean oil. Theoret Appl Genet.

[49] Liu Q, Xu W, Han S, Cao D, He X, Huang K, Mei X (2014). Production and optimization of a kiwi pectin methylesterase inhibitor in *Pichia pastoris* GS115. Food Sci Biotech.

[50] Mensink RPO, Temme EH, Hornstra G (1994). Dietary saturated and trans fatty acids and lipoprotein metabolism. Annl Med.

[51] Babiychuk E, Vandepoele K, Wissing J, Garcia-Diaz M, De Rycke R, Akbari H, Joubes J, Beeckman T, Jansch L, Frentzen M, Van Montaqu MCE, Kushnir S (2011). Plastic gene expression and plant development require a plastidic protein of the mitochondrial transcription termination factor family. Proc Nat Acad Sci..

[52] Wilson RF, Boerma H, Specht JE (2004). Soybeans: improvement, production and uses. Seed composition.

[53] Brachi B, Morris GP, Borevitz JO (2011). Genome-wide association studies in plants: the missing heritability is in the field. Genome Biol.

[54] Anderson CA, Pettersson FH, Clrke GM, Cardon LR, Morris AP, Zondervan ZT (2010). Data quality control in genetic case-control association studies. Nat Protoc.

[55] Oh S, Lee J, Kwon MS, Weir B, Ha K, Park T (2012). A novel method to identify high order gene-gene interactions in genome-wide association studies: gene-based MDR. BMC Bioinform.

[56] Zhang J, Singh A, Mueller D, Singh AK (2015). Genome-wide association and epistasis studies unravel the genetic architecture of sudden death syndrome resistance in soybean. The Plant J.

[57] Lu W, Wen Z, Li H, uan D, Li J, Zhang H, Huang Z, Cui S, Du W. Identification of the quantitative trait loci (QTL) underlying water soluble protein content in soybean. Theor Appl Genet. 2012; doi 10.1007/s00122-012-1990-8.. 10.1007/s00122-012-1990-8 23052024

[58] Reinprecht Y, Poysa VW, Yu K, Raican I, Ablett GR, Pauls KP (2006). Seed and agronomic QTL in low linolenic acid, lipoxygenase-free soybean (Glycine max (L.) Merrill) germplasm. Genome.

[59] Panthee DR, Pantalone VR, Saxton AM. Modifier QTL for fatty acid composition in soybean oil. Euphyt. 2006; doi: 10.1007/s10681-006-9179-3

[60] Bachlava E, Dewey R, Burton JW, Cardinal AJ (2009). Mapping and comparison of quantitative trait loci for oleic acid seed content in two segregating soybean populations. Crop Sci.

