# Supplementary material for: A genome-wide association study of seed composition traits in wild soybean (Glycine soja)
Source: BMC Genomics. 2017 Jan 5;18:18. doi: 10.1186/s12864-016-3397-4 (PMC5217241; doi:10.1186/s12864-016-3397-4)
Supplement: Additional file 1: — The country and province where the 570 accessions (PI) used in the analysis originated. Blank entries indicate unknown origins. (DOCX 37 kb) [file 12864_2016_3397_MOESM1_ESM.docx]

| **PI** | **Country** | **Province** |
| --- | --- | --- |
| PI163453 | China | Zhejiang |
| PI203246 | Japan | Fukuoka |
| PI245331 |  |  |
| PI339731 | Korea, South | Kangwon |
| PI339733 | Korea, South | Kangwon |
| PI339871A | Korea, South | Cheju |
| PI339871B | Korea, South | Cheju |
| PI339871C | Korea, South | Cheju |
| PI349647 | Korea, South | Kyonggi |
| PI366119 | Japan | Aichi |
| PI366124 | Japan | Kagawa |
| PI366125 | Japan | Saitama |
| PI378683 | Japan | Ishikawa |
| PI378684A | Japan | Saitama |
| PI378684B | Japan | Saitama |
| PI378685 | Japan | Ehime |
| PI378686A | Japan | Kochi |
| PI378686B | Japan | Kochi |
| PI378687A | Japan | Kumamoto |
| PI378687B | Japan | Kumamoto |
| PI378688 | Japan | Nara |
| PI378689 | Japan | Niigata |
| PI378690 | Japan | Fukuoka |
| PI378691 | Japan | Miyazaki |
| PI378694 |  |  |
| PI378695A |  |  |
| PI378695B |  |  |
| PI378696A |  |  |
| PI378696B |  |  |
| PI378697A | Japan | Aomori |
| PI378697B | Japan | Aomori |
| PI378698 | Japan | Yamanashi |
| PI378699A | Japan | Tokyo |
| PI378699B | Japan | Tokyo |
| PI378700 | Japan | Yamagata |
| PI378701A | Japan | Iwate |
| PI378701B | Japan | Iwate |
| PI393551 | Taiwan |  |
| PI407018 | Japan | Akita |
| PI407019 | Japan | Akita |
| PI407020 | Japan | Akita |
| PI407021 | Japan | Akita |
| PI407022 | Japan | Akita |
| PI407023 | Japan | Akita |
| PI407024 | Japan | Akita |
| PI407025 | Japan | Akita |
| PI407026 | Japan | Akita |
| PI407027 | Japan | Akita |
| PI407028 | Japan | Akita |
| PI407030 | Japan | Akita |
| PI407031 | Japan | Akita |
| PI407032A | Japan | Akita |
| PI407033 | Japan | Akita |
| PI407034 | Japan | Akita |
| PI407035 | Japan | Akita |
| PI407036 | Japan | Akita |
| PI407037 | Japan | Akita |
| PI407038 | Japan | Akita |
| PI407039 | Japan | Akita |
| PI407040 | Japan | Akita |
| PI407041 | Japan | Akita |
| PI407042 | Japan | Akita |
| PI407043 | Japan | Akita |
| PI407044 | Japan | Akita |
| PI407045 | Japan | Akita |
| PI407046 | Japan | Akita |
| PI407047 | Japan | Akita |
| PI407048 | Japan | Iwate |
| PI407049 | Japan | Iwate |
| PI407050 | Japan | Iwate |
| PI407051 | Japan | Iwate |
| PI407052 | Japan | Iwate |
| PI407053 | Japan | Nagano |
| PI407054 | Japan | Nagano |
| PI407055 | Japan | Shizuoka |
| PI407056 | Japan | Aichi |
| PI407057 | Japan | Aichi |
| PI407058 | Japan | Aichi |
| PI407059 | Japan | Aichi |
| PI407060 | Japan | Aichi |
| PI407061 | Japan | Aichi |
| PI407062 | Japan | Aichi |
| PI407063 | Japan | Aichi |
| PI407064 | Japan | Aichi |
| PI407065 | Japan | Aichi |
| PI407066 | Japan | Aichi |
| PI407067 | Japan | Aichi |
| PI407068 | Japan | Aichi |
| PI407069 | Japan | Aichi |
| PI407070 | Japan | Aichi |
| PI407071 | Japan | Aichi |
| PI407072 | Japan | Aichi |
| PI407073 | Japan | Aichi |
| PI407074 | Japan | Aichi |
| PI407075 | Japan | Aichi |
| PI407076 | Japan | Aichi |
| PI407077 | Japan | Aichi |
| PI407078 | Japan | Aichi |
| PI407079 | Japan | Aichi |
| PI407080 | Japan | Aichi |
| PI407081 | Japan | Aichi |
| PI407082 | Japan | Aichi |
| PI407083 | Japan | Aichi |
| PI407084 | Japan | Aichi |
| PI407085 | Japan | Aichi |
| PI407086 | Japan | Aichi |
| PI407087 | Japan | Hyogo |
| PI407088 | Japan | Hyogo |
| PI407089 | Japan | Hyogo |
| PI407090 | Japan | Hyogo |
| PI407091 | Japan | Hyogo |
| PI407092 | Japan | Hyogo |
| PI407093 | Japan | Hyogo |
| PI407094 | Japan | Hyogo |
| PI407095 | Japan | Hyogo |
| PI407096 | Japan | Hyogo |
| PI407097 | Japan | Hyogo |
| PI407098 | Japan | Hyogo |
| PI407100 | Japan | Hyogo |
| PI407101 | Japan | Hyogo |
| PI407102 | Japan | Hyogo |
| PI407103 | Japan | Hyogo |
| PI407104 | Japan | Hyogo |
| PI407105 | Japan | Hyogo |
| PI407107 | Japan | Hyogo |
| PI407108 | Japan | Hyogo |
| PI407109 | Japan | Hyogo |
| PI407110 | Japan | Hyogo |
| PI407111 | Japan | Hyogo |
| PI407112 | Japan | Hyogo |
| PI407114 | Japan | Hyogo |
| PI407115 | Japan | Hyogo |
| PI407116 | Japan | Hyogo |
| PI407117 | Japan | Hyogo |
| PI407118 | Japan | Hyogo |
| PI407119 | Japan | Hyogo |
| PI407120 | Japan | Hyogo |
| PI407121 | Japan | Hyogo |
| PI407122 | Japan | Hyogo |
| PI407124 | Japan | Hyogo |
| PI407125 | Japan | Hyogo |
| PI407126 | Japan | Okayama |
| PI407127 | Japan | Kumamoto |
| PI407128 | Japan | Kumamoto |
| PI407129 | Japan | Kumamoto |
| PI407130 | Japan | Kumamoto |
| PI407131 | Japan | Kumamoto |
| PI407132 | Japan | Kumamoto |
| PI407133 | Japan | Kumamoto |
| PI407135 | Japan | Kumamoto |
| PI407136 | Japan | Kumamoto |
| PI407138 | Japan | Kumamoto |
| PI407139 | Japan | Kumamoto |
| PI407140 | Japan | Kumamoto |
| PI407141 | Japan | Kumamoto |
| PI407142 | Japan | Kumamoto |
| PI407143 | Japan | Kumamoto |
| PI407144 | Japan | Kumamoto |
| PI407145 | Japan | Kumamoto |
| PI407146 | Japan | Kumamoto |
| PI407147 | Japan | Kumamoto |
| PI407148 | Japan | Kumamoto |
| PI407149 | Japan | Kumamoto |
| PI407150 | Japan | Kumamoto |
| PI407151 | Japan | Kumamoto |
| PI407152 | Japan | Kumamoto |
| PI407153 | Japan | Kumamoto |
| PI407154 | Japan | Kumamoto |
| PI407155 | Japan | Kumamoto |
| PI407157 | Japan | Chiba |
| PI407158 | Japan | Chiba |
| PI407159 | Korea, South | Kyonggi |
| PI407160 | Korea, South | Kyonggi |
| PI407161 | Korea, South | Kyonggi |
| PI407163 | Korea, South | Kyonggi |
| PI407164 | Korea, South | Kyonggi |
| PI407165 | Korea, South | Kyonggi |
| PI407167 | Korea, South | Kyonggi |
| PI407168 | Korea, South | Kyonggi |
| PI407170 | Korea, South | Kyonggi |
| PI407172 | Korea, South | Kyonggi |
| PI407174 | Korea, South | Kyonggi |
| PI407177 | Korea, South | Kyonggi |
| PI407178 | Korea, South | Kyonggi |
| PI407179 | Korea, South | Kyonggi |
| PI407180 | Korea, South | Kyonggi |
| PI407181 | Korea, South | Kyonggi |
| PI407183 | Korea, South | Kyonggi |
| PI407185 | Korea, South | Kyonggi |
| PI407186 | Korea, South | Kyonggi |
| PI407187 | Korea, South | Kyonggi |
| PI407188 | Korea, South | Kyonggi |
| PI407189 | Korea, South | Kyonggi |
| PI407190 | Korea, South | Kyonggi |
| PI407191 | Korea, South | Kyonggi |
| PI407192 | Korea, South | Kangwon |
| PI407193 | Korea, South | Kangwon |
| PI407194 | Korea, South | Kangwon |
| PI407198 | Korea, South | Kangwon |
| PI407199 | Korea, South | Kangwon |
| PI407201 | Korea, South | Kangwon |
| PI407202 | Korea, South | Kangwon |
| PI407203 | Korea, South | Kangwon |
| PI407204 | Korea, South | Kangwon |
| PI407206 | Korea, South | Kangwon |
| PI407207 | Korea, South | Kangwon |
| PI407208 | Korea, South | Chungchong Puk |
| PI407210 | Korea, South | Chungchong Puk |
| PI407211 | Korea, South | Chungchong Puk |
| PI407212 | Korea, South | Chungchong Puk |
| PI407213 | Korea, South | Chungchong Puk |
| PI407214 | Korea, South | Chungchong Puk |
| PI407215 | Korea, South | Chungchong Puk |
| PI407216 | Korea, South | Chungchong Puk |
| PI407218 | Korea, South | Chungchong Puk |
| PI407219 | Korea, South | Chungchong Puk |
| PI407220 | Korea, South | Chungchong Puk |
| PI407221 | Korea, South | Chungchong Puk |
| PI407222 | Korea, South | Chungchong Puk |
| PI407223 | Korea, South | Chungchong Puk |
| PI407224 | Korea, South | Chungchong Puk |
| PI407225 | Korea, South | Chungchong Nam |
| PI407226 | Korea, South | Chungchong Nam |
| PI407227 | Korea, South | Chungchong Nam |
| PI407228 | Korea, South | Chungchong Nam |
| PI407229 | Korea, South | Chungchong Nam |
| PI407231 | Korea, South | Chungchong Nam |
| PI407235 | Korea, South | Chungchong Nam |
| PI407236 | Korea, South | Chungchong Nam |
| PI407238 | Korea, South | Chungchong Nam |
| PI407239 | Korea, South | Chungchong Nam |
| PI407240 | Korea, South | Kyongsang Puk |
| PI407241 | Korea, South | Kyongsang Puk |
| PI407242 | Korea, South | Kyongsang Puk |
| PI407243 | Korea, South | Kyongsang Puk |
| PI407246 | Korea, South | Kyongsang Puk |
| PI407247 | Korea, South | Kyongsang Puk |
| PI407249 | Korea, South | Kyongsang Puk |
| PI407250 | Korea, South | Kyongsang Puk |
| PI407251 | Korea, South | Kyongsang Puk |
| PI407252 | Korea, South | Kyongsang Nam |
| PI407253 | Korea, South | Kyongsang Nam |
| PI407254 | Korea, South | Kyongsang Nam |
| PI407255 | Korea, South | Kyongsang Nam |
| PI407256 | Korea, South | Kyongsang Nam |
| PI407257 | Korea, South | Kyongsang Nam |
| PI407258 | Korea, South | Kyongsang Nam |
| PI407259 | Korea, South | Kyongsang Nam |
| PI407260 | Korea, South | Kyongsang Nam |
| PI407261 | Korea, South | Kyongsang Nam |
| PI407262 | Korea, South | Kyongsang Nam |
| PI407263 | Korea, South | Kyongsang Nam |
| PI407264 | Korea, South | Kyongsang Nam |
| PI407265 | Korea, South | Kyongsang Nam |
| PI407266 | Korea, South | Kyongsang Nam |
| PI407267 | Korea, South | Kyongsang Nam |
| PI407268 | Korea, South | Kyongsang Nam |
| PI407270 | Korea, South | Kyongsang Nam |
| PI407271 | Korea, South | Cholla Puk |
| PI407272 | Korea, South | Cholla Puk |
| PI407273 | Korea, South | Cholla Puk |
| PI407274 | Korea, South | Cholla Puk |
| PI407276 | Korea, South | Kyonggi |
| PI407280 |  |  |
| PI407281 |  |  |
| PI407282 |  |  |
| PI407283 |  |  |
| PI407284 |  |  |
| PI407285 | Japan | Kanagawa |
| PI407286 | Japan | Kanagawa |
| PI407287 | Japan | Kanagawa |
| PI407300 | China | Jiangsu |
| PI407301 | China | Jiangsu |
| PI407302 | China | Jiangsu |
| PI407303 | China | Jiangsu |
| PI407304 | China | Shanghai |
| PI407305 | China | Shanghai |
| PI407306 | China | Shanghai |
| PI407307 | China | Shanghai |
| PI407308 | Korea, South | Kyonggi |
| PI407309A | Korea, South | Kyonggi |
| PI407309B | Korea, South | Kyonggi |
| PI407310 | Korea, South | Kyonggi |
| PI407311 | Korea, South | Kyonggi |
| PI407312 | Korea, South | Chungchong Puk |
| PI407313 | Korea, South | Chungchong Puk |
| PI407314 | Korea, South | Chungchong Puk |
| PI407315 | Korea, South | Chungchong Puk |
| PI407316A | Korea, South | Chungchong Puk |
| PI407316B | Korea, South | Chungchong Puk |
| PI407317 | Korea, South | Chungchong Nam |
| PI407318A | Korea, South | Chungchong Puk |
| PI407318B | Korea, South | Chungchong Puk |
| PI407319 | Korea, South | Chungchong Puk |
| PI407322 | Korea, South | Chungchong Puk |
| PI424008B | Korea, South | Kyonggi |
| PI424009 | Korea, South | Kyonggi |
| PI424010 | Korea, South | Kyonggi |
| PI424012 | Korea, South | Kyonggi |
| PI424013 | Korea, South | Kyonggi |
| PI424014 | Korea, South | Kyonggi |
| PI424015 | Korea, South | Kyonggi |
| PI424016 | Korea, South | Kyonggi |
| PI424017A | Korea, South | Kyonggi |
| PI424017B | Korea, South | Kyonggi |
| PI424018 | Korea, South | Kyonggi |
| PI424019 | Korea, South | Kyonggi |
| PI424020A | Korea, South | Kyonggi |
| PI424020B | Korea, South | Kyonggi |
| PI424021A | Korea, South | Kyonggi |
| PI424021B | Korea, South | Kyonggi |
| PI424022A | Korea, South | Kyonggi |
| PI424022B | Korea, South | Kyonggi |
| PI424023 | Korea, South | Kyonggi |
| PI424025A | Korea, South | Kyonggi |
| PI424025B | Korea, South | Kyonggi |
| PI424025C | Korea, South | Kyonggi |
| PI424026 | Korea, South | Kyonggi |
| PI424027A | Korea, South | Kyonggi |
| PI424027B | Korea, South | Kyonggi |
| PI424028 | Korea, South | Kyonggi |
| PI424029 | Korea, South | Kyonggi |
| PI424030 | Korea, South | Kyonggi |
| PI424031 | Korea, South | Kyonggi |
| PI424035 | Korea, South | Kyonggi |
| PI424039A | Korea, South | Kyonggi |
| PI424039B | Korea, South | Kyonggi |
| PI424040 | Korea, South | Kyonggi |
| PI424041 | Korea, South | Kyonggi |
| PI424042 | Korea, South | Kyonggi |
| PI424043A | Korea, South | Kyonggi |
| PI424043B | Korea, South | Kyonggi |
| PI424044 | Korea, South | Kyonggi |
| PI424045 | Korea, South | Kyonggi |
| PI424046A | Korea, South | Kyonggi |
| PI424046B | Korea, South | Kyonggi |
| PI424047 | Korea, South | Kyonggi |
| PI424048 | Korea, South | Kyonggi |
| PI424049 | Korea, South | Kyonggi |
| PI424050 | Korea, South | Kyonggi |
| PI424052 | Korea, South | Kyonggi |
| PI424053 | Korea, South | Kyonggi |
| PI424054A | Korea, South | Kyonggi |
| PI424054B | Korea, South | Kyonggi |
| PI424055 | Korea, South | Kyonggi |
| PI424056 | Korea, South | Kangwon |
| PI424057 | Korea, South | Kangwon |
| PI424058 | Korea, South | Kangwon |
| PI424059A | Korea, South | Kangwon |
| PI424059B | Korea, South | Kangwon |
| PI424060 | Korea, South | Kangwon |
| PI424061A | Korea, South | Kangwon |
| PI424061B | Korea, South | Kangwon |
| PI424062 | Korea, South | Kangwon |
| PI424064 | Korea, South | Kangwon |
| PI424065 | Korea, South | Kangwon |
| PI424066 | Korea, South | Kangwon |
| PI424069 | Korea, South | Kangwon |
| PI424070A | Korea, South | Kangwon |
| PI424070B | Korea, South | Kangwon |
| PI424072A | Korea, South | Kangwon |
| PI424072B | Korea, South | Kangwon |
| PI424073 | Korea, South | Kangwon |
| PI424074 | Korea, South | Kangwon |
| PI424075 | Korea, South | Kangwon |
| PI424076A | Korea, South | Kangwon |
| PI424076B | Korea, South | Kangwon |
| PI424077 | Korea, South | Kangwon |
| PI424080 | Korea, South | Kangwon |
| PI424081A | Korea, South | Kangwon |
| PI424081B | Korea, South | Kangwon |
| PI424082 | Korea, South | Kangwon |
| PI424083A | Korea, South | Kangwon |
| PI424083B | Korea, South | Kangwon |
| PI424084 | Korea, South | Kangwon |
| PI424085A | Korea, South | Kangwon |
| PI424085B | Korea, South | Kangwon |
| PI424086 | Korea, South | Chungchong Puk |
| PI424087 | Korea, South | Chungchong Puk |
| PI424089 | Korea, South | Chungchong Puk |
| PI424090 | Korea, South | Chungchong Puk |
| PI424093 | Korea, South | Chungchong Puk |
| PI424095 | Korea, South | Chungchong Puk |
| PI424096 | Korea, South | Chungchong Nam |
| PI424097 | Korea, South | Kyongsang Puk |
| PI424098 | Korea, South | Kyongsang Puk |
| PI424099A | Korea, South | Kyongsang Puk |
| PI424099B | Korea, South | Kyongsang Puk |
| PI424100A | Korea, South | Kyongsang Puk |
| PI424100B | Korea, South | Kyongsang Puk |
| PI424101 | Korea, South | Kyongsang Puk |
| PI424102A | Korea, South | Kyongsang Puk |
| PI424102B | Korea, South | Kyongsang Puk |
| PI424103A | Korea, South | Kyongsang Puk |
| PI424104 | Korea, South | Kyongsang Puk |
| PI424105 | Korea, South | Kyongsang Puk |
| PI424106B | Korea, South | Kyongsang Puk |
| PI424107A | Korea, South | Kyongsang Puk |
| PI424107B | Korea, South | Kyongsang Puk |
| PI424108 | Korea, South | Kyongsang Puk |
| PI424109A | Korea, South | Kyongsang Puk |
| PI424109B | Korea, South | Kyongsang Puk |
| PI424110 | Korea, South | Kyongsang Puk |
| PI424111 | Korea, South | Kyongsang Puk |
| PI424112 | Korea, South | Kyongsang Puk |
| PI424113 | Korea, South | Kyongsang Puk |
| PI424114A | Korea, South | Kyongsang Puk |
| PI424114B | Korea, South | Kyongsang Puk |
| PI424115A | Korea, South | Kyongsang Puk |
| PI424115B | Korea, South | Kyongsang Puk |
| PI424117 | Korea, South | Kyongsang Puk |
| PI424118 | Korea, South | Kyongsang Puk |
| PI424119 | Korea, South | Kyongsang Puk |
| PI424120 | Korea, South | Kyongsang Nam |
| PI424121 | Korea, South | Kyongsang Nam |
| PI424122 | Korea, South | Kyongsang Nam |
| PI424123 | Korea, South | Kyongsang Nam |
| PI424124 | Korea, South | Kyongsang Nam |
| PI424125 | Korea, South | Cholla Puk |
| PI424126 | Korea, South | Cholla Puk |
| PI424127A | Korea, South | Cholla Puk |
| PI424127B | Korea, South | Cholla Puk |
| PI424128A | Korea, South | Cholla Puk |
| PI424128B | Korea, South | Cholla Puk |
| PI424129 | Korea, South | Cholla Puk |
| PI424130 | Korea, South | Cholla Puk |
| PI464934 |  |  |
| PI464935 |  |  |
| PI464936A |  |  |
| PI464936B |  |  |
| PI464937A |  |  |
| PI464937B |  |  |
| PI464939A |  |  |
| PI464939B |  |  |
| PI483465 | China | Shaanxi |
| PI483466 | China | Shandong |
| PI483467 | China | Henan |
| PI483468A | China | Henan |
| PI486220 | Japan | Shizuoka |
| PI487428 | Japan | Iwate |
| PI487429 | Japan | Kanagawa |
| PI487430 | Japan | Hokkaido |
| PI487431 | Japan | Kagoshima |
| PI504286 | Korea, South | Chungchong Puk |
| PI504287B | Japan | Iwate |
| PI504289 | Japan | Iwate |
| PI504290 | Japan | Iwate |
| PI507580 | Japan | . |
| PI507582 | Japan | Aomori |
| PI507583 | Japan | Aomori |
| PI507585 | Japan | Aomori |
| PI507586 | Japan | Aomori |
| PI507587 | Japan | Aomori |
| PI507588 | Japan | Aomori |
| PI507590A | Japan | Akita |
| PI507590B | Japan | Akita |
| PI507593 | Japan | Fukushima |
| PI507594B | Japan | Fukushima |
| PI507595 | Japan | Niigata |
| PI507596 | Japan | Niigata |
| PI507597 | Japan | Niigata |
| PI507598 | Japan | Toyama |
| PI507599 | Japan | Ibaraki |
| PI507600 | Japan | Ibaraki |
| PI507601A | Japan | Ibaraki |
| PI507601B | Japan | Ibaraki |
| PI507602 | Japan | Ibaraki |
| PI507603 | Japan | Ibaraki |
| PI507604 | Japan | Ibaraki |
| PI507605 | Japan | Ibaraki |
| PI507606 | Japan | Tochigi |
| PI507607 | Japan | Tochigi |
| PI507608 | Japan | Tochigi |
| PI507609 | Japan | Tochigi |
| PI507611 | Japan | Tochigi |
| PI507612 | Japan | Tochigi |
| PI507613 | Japan | Gunma |
| PI507614A | Japan | Nagano |
| PI507614B | Japan | Nagano |
| PI507616 | Japan | Nagano |
| PI507617 | Japan | Nagano |
| PI507618 | Japan | Nagano |
| PI507619A | Japan | Nagano |
| PI507619B | Japan | Nagano |
| PI507619C | Japan | Nagano |
| PI507620 | Japan | Nagano |
| PI507621 | Japan | Nagano |
| PI507622 | Japan | Nagano |
| PI507623 | Japan | Tokyo |
| PI507624 | Japan | Gifu |
| PI507625 | Japan | Fukui |
| PI507626 | Japan | Aichi |
| PI507627 | Japan | Aichi |
| PI507628 | Japan | Aichi |
| PI507629 | Japan | Shizuoka |
| PI507630 | Japan | Nara |
| PI507631 | Japan | Nara |
| PI507632 | Japan | Nara |
| PI507633 | Japan | Kyoto |
| PI507634 | Japan | Hyogo |
| PI507635 | Japan | Hyogo |
| PI507636 | Japan | Hyogo |
| PI507637 | Japan | Hyogo |
| PI507638 | Japan | Hyogo |
| PI507639 | Japan | Hyogo |
| PI507640 | Japan | Hyogo |
| PI507641 | Japan | Hyogo |
| PI507642 | Japan | Hyogo |
| PI507645 | Japan | Tokushima |
| PI507646 | Japan | Hiroshima |
| PI507647 | Japan | Okayama |
| PI507648 | Japan | Okayama |
| PI507649 | Japan | Okayama |
| PI507650 | Japan | Tottori |
| PI507651 | Japan | Yamaguchi |
| PI507652 | Japan | Yamaguchi |
| PI507653 | Japan | Fukuoka |
| PI507654 | Japan | Saga |
| PI507655 | Japan | Nagasaki |
| PI507656 | Japan | Nagasaki |
| PI507657 | Japan | Kagoshima |
| PI507658 | Japan | Kagoshima |
| PI507659 | Japan | Kagoshima |
| PI507660 | Japan | Kagoshima |
| PI507661 | Japan | Kagoshima |
| PI507662 | Japan | Kagoshima |
| PI507663 | Japan | Kagoshima |
| PI507664 | Japan | Kumamoto |
| PI507665 | Japan | Kumamoto |
| PI507666 | Japan | Kumamoto |
| PI507667 | Japan | Kumamoto |
| PI507668 | Japan | Kumamoto |
| PI507669 | Japan | Kumamoto |
| PI518280 | Taiwan | . |
| PI518281 |  |  |
| PI518282 |  |  |
| PI562531 | Korea, South | Kyonggi |
| PI562532 | Korea, South | Kyonggi |
| PI562533 | Korea, South | Kyonggi |
| PI562534 | Korea, South | Kyonggi |
| PI562535 | Korea, South | Kyonggi |
| PI562536 | Korea, South | Kyonggi |
| PI562537 | Korea, South | Kyonggi |
| PI562538 | Korea, South | Kyonggi |
| PI562539 | Korea, South | Kyonggi |
| PI562540 | Korea, South | Kyonggi |
| PI562541 | Korea, South | Kyonggi |
| PI562542 | Korea, South | Kyonggi |
| PI562543 | Korea, South | Chungchong Nam |
| PI562544 | Korea, South | Chungchong Nam |
| PI562545 | Korea, South | Chungchong Nam |
| PI562546 | Korea, South | Chungchong Nam |
| PI562547 | Korea, South | Chungchong Nam |
| PI562548 | Korea, South | Chungchong Nam |
| PI562549 | Korea, South | Chungchong Nam |
| PI562551 | Korea, South | Chungchong Nam |
| PI562552 | Korea, South | Chungchong Nam |
| PI562553 | Korea, South | Chungchong Nam |
| PI562554 | Korea, South | Chungchong Nam |
| PI562555 | Korea, South | Chungchong Nam |
| PI562556 | Korea, South | Cholla Puk |
| PI562557 | Korea, South | Cholla Puk |
| PI562558 | Korea, South | Cholla Puk |
| PI562559 | Korea, South | Cholla Puk |
| PI562561 | Korea, South | Cholla Puk |
| PI562562 | Korea, South | Cholla Puk |
| PI562563 | Korea, South | Cholla Puk |
| PI562566 | Korea, South | Cholla Puk |
| PI562567 | Korea, South | Cholla Puk |
| PI562568 | Korea, South | Cholla Puk |
