# Supplementary material for: A genome-wide association study of seed composition traits in wild soybean (Glycine soja)
Source: BMC Genomics. 2017 Jan 5;18:18. doi: 10.1186/s12864-016-3397-4 (PMC5217241; doi:10.1186/s12864-016-3397-4)
Supplement: Supplementary file 2 [file 12864_2016_3397_MOESM2_ESM.docx]

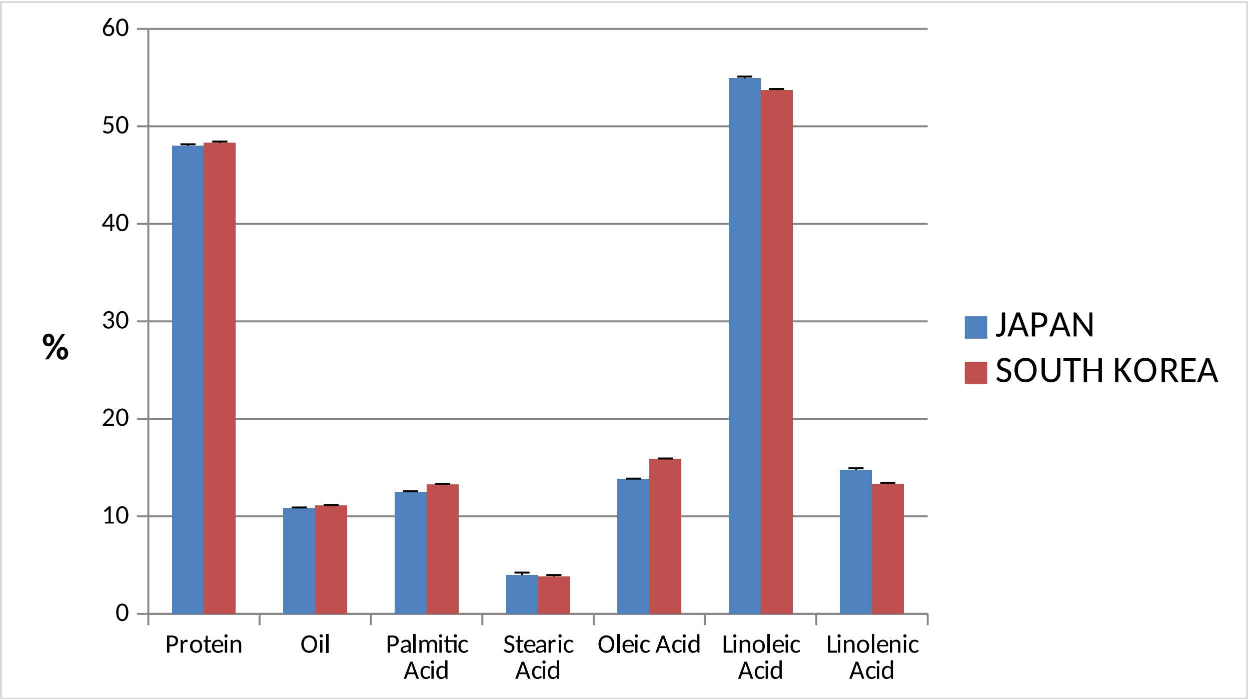

60
50
40
30
20
10
0
%
Protein
Oil
Palmitic Acid
Stearic Acid
Oleic Acid
Linoleic Acid
Linolenic Acid
JAPAN
SOUTH KOREA
