# Supplementary material for: A genome-wide association study of seed composition traits in wild soybean (Glycine soja)
Source: BMC Genomics. 2017 Jan 5;18:18. doi: 10.1186/s12864-016-3397-4 (PMC5217241; doi:10.1186/s12864-016-3397-4)
Supplement: Supplementary file 3 [file 12864_2016_3397_MOESM3_ESM.docx]

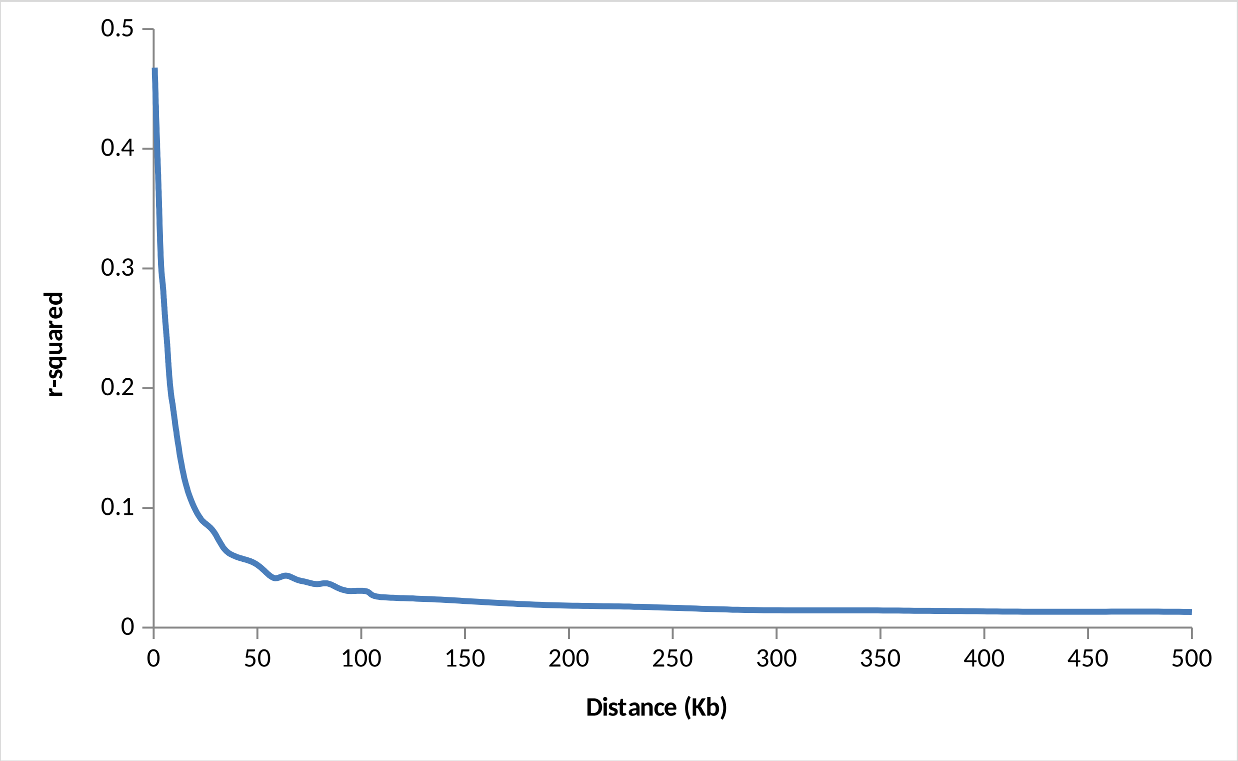
0.5
0.4
0.3
0.2
0.1
0
r-squared
0
50
100
150
200
250
300
350
400
450
500
Distance (Kb)
