# Supplementary material for: A genome-wide association study of seed composition traits in wild soybean (Glycine soja)
Source: BMC Genomics. 2017 Jan 5;18:18. doi: 10.1186/s12864-016-3397-4 (PMC5217241; doi:10.1186/s12864-016-3397-4)
Supplement: Supplementary file 4 [file 12864_2016_3397_MOESM4_ESM.docx]

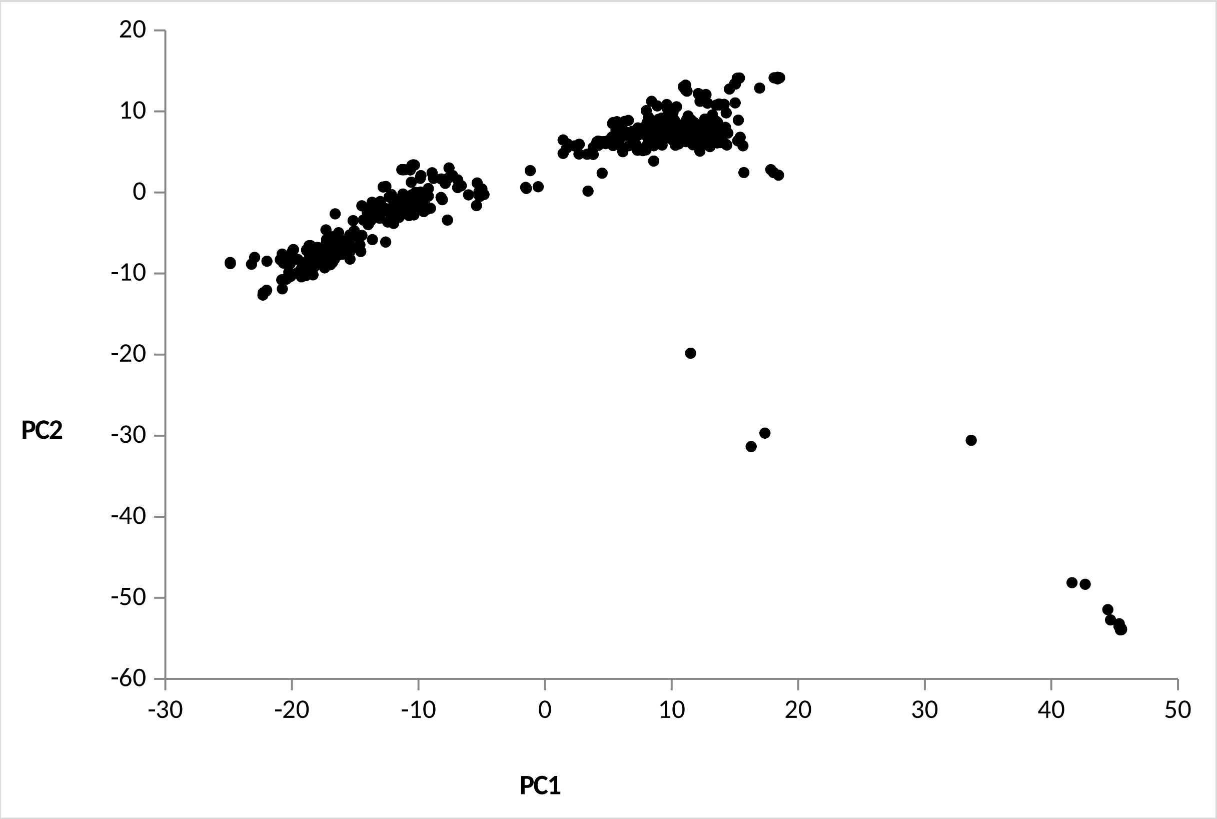
20
10
0
-10
-20
-30
-40
-50
-60
PC2
-30
-20
-10
0
10
20
30
40
50
PC1
